# A global analysis of bioeconomy visions in governmental bioeconomy strategies

**DOI:** 10.1007/s13280-023-01958-6

**Published:** 2023-12-27

**Authors:** Maria Proestou, Nicolai Schulz, Peter H. Feindt

**Affiliations:** Thaer-Institut for Agricultural and Horticultural Sciences, Agricultural and Food Policy Group, Humboldt-Universität zu Berlin, Unter den Linden 6, 10099 Berlin, Germany

**Keywords:** Bio-based economy, Bioecology, Bioresource, Biotechnology, Policy goals, Vision typology

## Abstract

Against the background of climate change and scarce non-renewable resources, transforming the fossil-based toward a bio-based economy is considered crucial for sustainable development. Numerous countries have released governmental strategies outlining their bioeconomy visions. This study examines the bioeconomy visions presented in 78 policy documents from 50 countries worldwide, building on earlier vision typologies. Through qualitative content analysis, 227 distinct policy goals were identified and analyzed. Descriptive statistics were used to determine the salience of specific goals, overarching goal categories, and distinct bioeconomy visions: bioresource, biotechnology, and bioecology visions. The results reveal that goals and visions prioritize economic growth, while environmental considerations are less salient. The bioresource vision emerges as the globally dominant perspective, while the bioecology and biotechnology visions have lower salience. These findings deepen our comprehension of current bioeconomy policies and emphasize the need for critical research on bioeconomy visions and their implications for public policy.

**Supplementary Information:**

The online version contains supplementary material available at 10.1007/s13280-023-01958-6.

## Introduction

In recent years, the concept of the bioeconomy has gained global prominence as a potential driver of sustainable economic growth. Generally understood as the range of economic activities based on the use of biological processes and renewable resources for agricultural and industrial purposes (German Bioeconomy Council 2018), it has triggered a wave of expectations and ambitions in politics, business, and academia. The concept of the bioeconomy is associated with transformative ambitions to replace fossil fuels with renewable resources, biomaterials, bioenergy, and biofuels in closed material cycles (Staffas et al. 2013; Scarlat et al. 2015; Murray et al. 2017). A rapidly growing number of national and international organizations and actors see the transformation to a sustainable bioeconomy as an important approach to addressing the major problems and conflicts of our time, such as food security, poverty, climate change, health risks, sustainable energy supply and biodiversity conservation (Philp 2018; IACGB 2020). In this context, bioeconomy-related policy statements have proliferated globally, as evidenced by the widespread adoption of national government bioeconomy-related policies or strategies in about 50 countries over the past 15 years (Meyer 2017; Dietz et al. 2018).

The bioeconomy, however, is a multifaceted and ambiguous concept. Its precise meaning and scope remains unsettled (Bugge et al. 2016) and it has become the object of political contestation (Vivien et al. 2019; Eversberg et al. 2023), with debates on various sustainability-related issues (e.g., the food-energy dilemma, the environment–development nexus, and the challenge of society technologization).^1^ To better understand the contours, different facets, and underlying ambitions of the concept, existing scholarship has provided valuable research and typologies of bioeconomy visions, i.e., more or less coherent sets of cognitive and normative ideas about the future shape of the bioeconomy and its purpose (Bugge et al. 2016; Hausknost et al. 2017; Dietz et al. 2018; Vivien et al. 2019). The study of bioeconomy visions is particularly important in relation to bioeconomy policy documents, as these form the basis for the selection and design of policy instruments, which in turn determine the pace, scope, and direction of the development of the bioeconomy.

Critically, however, previous studies have analyzed bioeconomy policy visions only at a highly aggregated and geographically limited scale. To date, no study has unpacked the key components of bioeconomy visions in related policy documents around the world. Furthermore, there is a lack of research that examines changes in the salience of different bioeconomy visions over time and variations across countries of different economic status. Filling these research gaps is important because it helps to provide researchers, civil society actors, and policymakers with a deeper, more comprehensive, and comparable understanding of the status and dynamics of governments’ intentions to promote, legitimize, and capitalize on the bioeconomy.

To address these critical gaps in the literature, our paper conducts a comprehensive analysis of bioeconomy visions and goals as articulated in 78 governmental bioeconomy policy documents from around the world. Consistent with previous research (Bugge et al. 2016), our focus is on the goals articulated in these documents, as they often serve as practical steps that reveal latent, if occasionally elusive, visions. Specifically, we employ a qualitative content analysis to identify the stated policy goals, group them into overarching categories, and link them to three main bioeconomy visions commonly identified in the literature. We then carefully examine both the prominence of these goals and visions and analyze how they have evolved across different temporal and spatial dimensions. As the most comprehensive and granular analysis of bioeconomy visions to date, this study provides new insights for the study of bioeconomy visions and strengthens its scientific foundation.

In the following sections, we will first define the concept of policy visions within the context of the bioeconomy, drawing upon existing typologies of bioeconomy visions. We then explain our methodology, detailing our case selection, and the operationalization of these visions. In the fourth section, we present and discuss our findings on the importance and orientation of visions in bioeconomy policies. Finally, we reflect on the limitations of our study and outline directions for future research.

## Conceptualizing bioeconomy visions

While the ‘utilization, management, and exploitation of biological processes and renewable resources for agricultural and industrial purposes’ (German Bioeconomy Council 2018) have long been an integral part of any economy, the current bioeconomy discourse is characterized by future-oriented statements along three dimensions: an intensification in the use of biological processes, a shift in the resource base of the entire economy toward renewable resources, and a broadening of the scope of applications. This multi-dimensionality provides space for different visions of the bioeconomy. D’Amato et al. (2017) describe the bioeconomy as focused on biomass and biotechnology applications, Meyer (2017) focuses on technology- and biomass-oriented visions and definitions, and Dietz et al. (2018) differentiate between bioresource-based and technology-intensive transformation pathways. Broader typologies are offered by Bugge et al. (2016), who, based on a systematic literature review, distinguish between biotechnology, bio-resource and bioecology visions. Similarly, Vivien et al. (2019) differentiate between ecological economy-oriented, science (biotechnology)-based and biomass-focused bioeconomy narratives. From a political ecology perspective, Hausknost et al. (2017) reconstruct four competing techno-political narratives of the bioeconomy which focus on industrial biotechnology, agroecology, sufficiency, and capitalist growth.

Representing the most comprehensive synthesis of these earlier works, we follow Bugge et al. (2016) by distinguishing three types of bioeconomy visions. First, *bio-technology visions* characterize a bioeconomy that focuses on economic growth and job creation through technological innovation, genetic engineering, commercialization of research and technology, and a focus on life sciences and health applications (Bugge et al. 2016; Vivien et al. 2019). Second, *bioresource visions* focus on the efficient production and use of biomass. At the heart of this vision are new crops, new products, and value chains, the closing of material cycles, waste processing, linking agriculture with industrial and energy production, and rural development. Similar to the biotechnology vision, the bio-resource vision emphasizes the technological development of new products for economic growth. At the same time, it addresses land use issues by focusing on the cascading use of biomass through biorefining (Bugge et al. 2016; Dietz et al. 2018; Vivien et al. 2019). Third, *bioecology visions* focus on the sustainable use of natural resources through agro-ecological approaches, high-quality biomass, and products with territorial identity, circular economy at regional scale, conservation of ecosystems and biodiversity, sustainability, and societal participation in the bioeconomy transition processes. In these visions, socio-ecological resilience, bioethics, and social inequalities are prominent topics (Bugge et al. 2016; Meyer 2017).

Governmental bioeconomy policies and strategy documents articulate bioeconomy visions in their goals and stated aims (Bugge et al. 2016; Dietz et al. 2018) and endow them with the authority, legitimacy, and potentially the resources of the state apparatus (Feindt et al. 2020a, b). They hence deserve particular scrutiny. Previous studies have identified bioeconomy visions in scientific publications (Bugge et al. 2016; D’Amato et al. 2017; Vivien et al. 2019; Holmgren et al. 2020; Ranacher et al. 2020), newspaper articles (Sanz-Hernández et al. 2020), population surveys (Eversberg and Fritz 2022), or in stakeholder interviews (Vivien et al. 2019). Analyses of bioeconomy visions in policy documents were based on either selective (Hausknost et al. 2017) or European-centric (Meyer 2017) samples. In a more inclusive and systematic approach, Dietz et al. (2018) examined the objectives and instruments of 41 national bioeconomy-related policies, but focused on the policies’ sustainability dimensions. A more encompassing analysis of governmental bioeconomy visions across all 50 countries with bioeconomy-related policies is lacking.

## Materials and methods

**Fig. 1:**
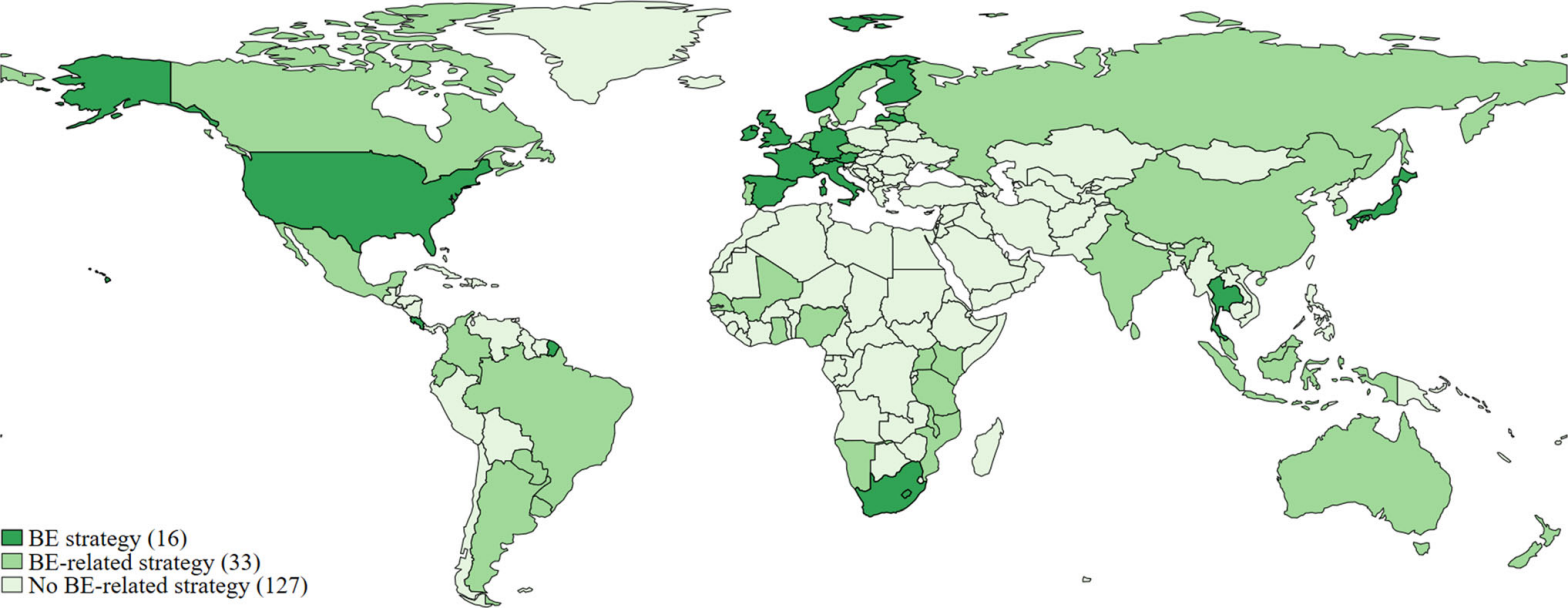
Geographical coverage of coded bio-related policy documents

To identify the visions articulated in bioeconomy-related policy documents around the globe, we conducted a systematic qualitative-quantitative content analysis of 78 policy documents from 50 countries (mapped in Fig. 1). Coded text segments were aggregated into document-level vision categories, and their patterns were mapped with descriptive statistics. The following sections explain the document selection criteria and the construction and operationalization of the analytical categories.

### Case selection

Our aim was to analyze all documents that most directly and comprehensively capture the contemporary bioeconomy orientation in a country. This required an inventory of all bioeconomy-related policy documents. Drawing on lists compiled by the 2018 and 2020 Global Bioeconomy Reports (IACGB 2020), Dietz et al. (2018), and our own research,^2^ we identified a total of 288 potentially relevant bioeconomy or bioeconomy-related policy documents. Since most of these documents did not represent the most direct or recent characterization of a country’s current bioeconomy policy, we further reduced this list in two steps. First, 16 countries have published bioeconomy strategies, i.e., strategies explicitly dedicated to promoting the bioeconomy in its entirety. In these cases, we exclusively analyzed a country’s most recent bioeconomy strategy, since it is the most direct, comprehensive, and effective representation of a country’s prevailing bioeconomy vision. Second, for countries that did not publish a bioeconomy strategy, we included all relevant bioeconomy-related documents in our analysis. We considered documents to be bioeconomy-related if their focus was directly related to at least one of the main areas of the bioeconomy, i.e., biotechnology, biomass, biofuels, biorefineries, bioindustries, bioenergy, the blue economy, or the circular economy. If a country had several strategies per area (e.g., two biofuel strategies), only the most recent one was selected.

In total, the sample includes 78 bioeconomy policy documents from 50 countries (see Fig. 1). Fifty-eight of them are from high- or upper-middle-income countries. Out of 16 bioeconomy strategies, only three were produced by governments outside the OECD. Besides bioeconomy strategies, biotechnology (19) and bioenergy (16) policies are the most common types of documents in our sample (cf. Appendix S1). More than three-quarters (61) of our sample documents were published after 2010, and exactly half (39) after 2015, with the sample cut-off year being 2020 (cf. Appendix S2).

### Operationalization of visions

Stated policy goals and aims are the most direct representation of a policy document’s vision. Therefore, to capture the visions of bioeconomy-related policy documents, we identified, coded, and categorized each document’s policy goals in several steps. First, qualitative content analysis (Neuendorf 2017; Krippendorff 2019; Mayring 2022) was applied to the entire text corpus to inductively generate a comprehensive list of all stated goals.^3^ These were then reviewed and combined into a structured coding scheme consisting of 227 goal-related codes. Using this scheme, we then systematically coded all documents using the qualitative content analysis software MAXQDA.^4^

We then categorized the 227 distinct goals into distinct vision categories. First, using our own approach, each goal was assigned to one (and in rare cases, two)^5^ of five distinct categories: “Economic,” “Environmental,” “Social,” “Political,” and “Research, Innovation & Technology.” To allow for more detailed analysis, each of these categories was further disaggregated into up to sixteen subcategories, which are described in the next section. Second, we subsumed our codes under the three bioeconomy visions derived from previous studies (bioresource, biotechnology, and bioecology). For this purpose, we systematically reviewed the core vision literature, extracted phrases and keywords that describe each vision type (Appendix S3), then checked each of the identified descriptions to determine which codes in our coding scheme matched them (Appendix S4), and subsequently assigned matching codes to the respective vision type (Appendix S5).^6^

Finally, in order to compare the salience of these respective categories, we created variables that indicate the percentage of a document’s goal-coded text that is coded with each of the specific vision categories and types (i.e., text shares that did not contain any goal statements are excluded from the denominator).^7^ To analyze the salience of subcategories, the text share of the respective main category serves as the denominator for our calculations. To assess the salience of different vision categories in the sample, we use simple descriptive statistics, mostly presented below in the form of bar charts and scatter plots.

## Results and discussion

This section is divided into two parts. First, we present and explore the relative salience of goals within categories and subcategories, including analyses across income groups and over time. Second, we assess the salience of the three different vision types, both in aggregate and across our 78 policy documents.

### Bioeconomy policy goals across categories

**Fig. 2:**
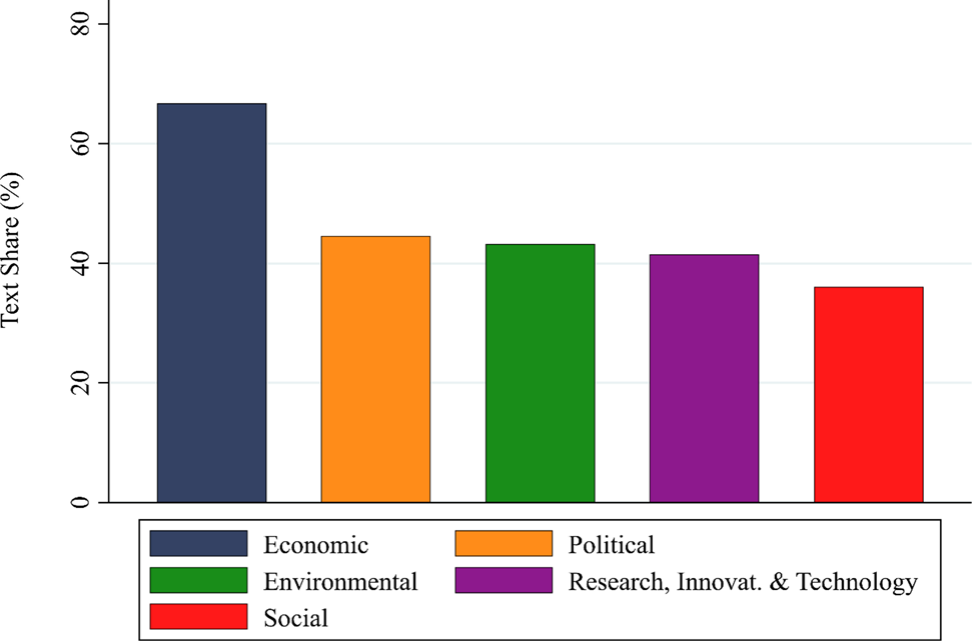
Text share of goal categories within goal-related text share in the sample (in percent)

First, to study the importance of different visions in bioeconomy policy documents, we turn to the analysis of our categorical system. Figure 2 depicts the average text share of each category within the total goal-coded text. Several results emerge. First, the economic category is by far the most salient category, present in 66.7% of all goal-related text. Second, the salience of the political, environmental, and research goal categories is similar at lower levels (at 44.5%, 43.2%, and 41.5% of the goal-related text share, respectively). The social category is least salient (at 36.1%).

The relative importance of economic versus environmental goals has been a central focus for social scientists studying bioeconomy discourses. The strong emphasis on economic goals is consistent with previous research that has described the dominance of economic considerations in bioeconomy policymaking (Hausknost et al. 2017; Meyer 2017; Böcher et al. 2020; D’Amato et al. 2020; Sanz-Hernández et al. 2020; Vogelpohl and Töller 2021; Eversberg et al. 2023; Vogelpohl 2023). The main reason for this relative dominance can likely be traced back to the initial motivation behind the creation and promotion of the bioeconomy in its current dominant ‘promissory’ framing in the late 1990s and 2000s (Eversberg et al. 2023). While the concept was also seen as an approach to addressing climate change and environmental degradation (Birch 2006; Kleinschmit et al. 2014), the dominant driver in policy discourses was the notion that a “life science revolution” and the perceived substantial growth potential of biotechnology would act as a new engine for economic growth (Benner and Löfgren 2007; OECD 2009; Petersen and Krisjansen 2015; Backhouse et al. 2021b; Ramcilovic-Suominen et al. 2022; Eversberg et al. 2023). Thus, the initial vision of bioeconomy policymaking revolved around biotechnology-driven economic growth with the added benefit of improved environmental sustainability.

**Fig. 3:**
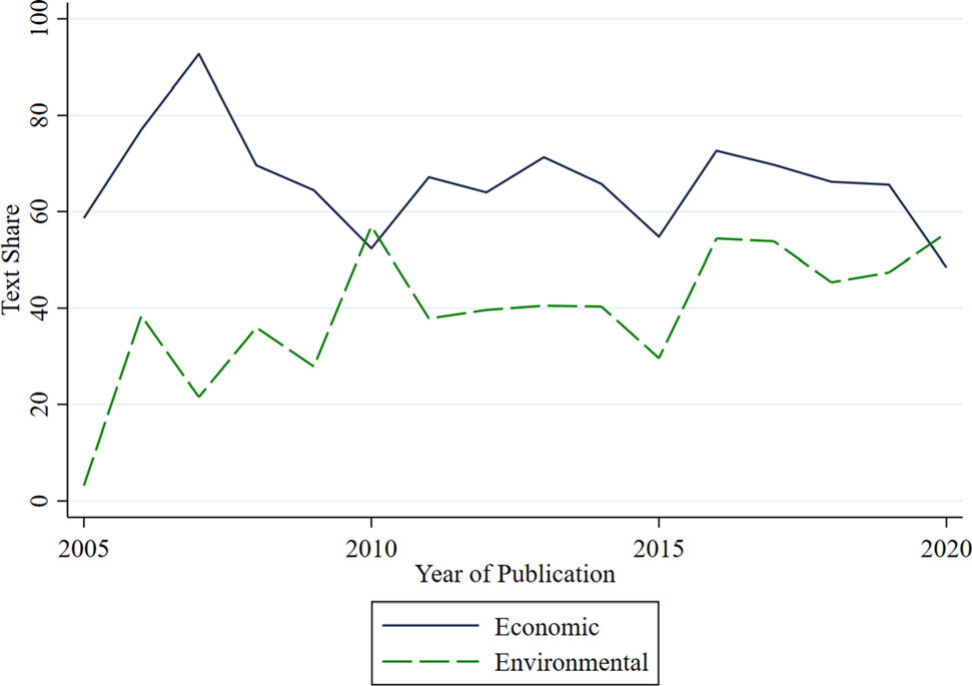
The relative text share of economic and environmental goals over time (in percent)

Importantly, our data reveal a shift in this economy-centered framing in the second half of the 2010s. As shown in Fig. 3, the gap between the prominence of economic and environmental goals narrowed significantly over the last 5 years of our sample. Prior to 2016, economic goals were on average 1.9 times more salient than environmental goals. This ratio decreased to 1.3 times in the following years. Notably, in the five strategies published in 2020 (the last year of our sample), the average proportion of text devoted to environmental goals actually exceeded that of economic goals. This shift is primarily driven by an increase in the prominence of environmental goals, which increased by nearly 14% compared to the pre-2016 period, while economic goals decreased by only 1%.

This transition to a greater focus on environmental goals is consistent with earlier findings in the literature. In particular, Eversberg et al. (2023) identify two significant developments in the mid-2010s. First, the adoption of the Sustainable Development Goals (SDGs) in September 2015, which emphasized the interconnectedness of environmental and economic aspects of sustainable development, likely provided an impetus for a more environmental-oriented bioeconomy policy framework. Indeed, we find that 48% of policy documents published since 2016 explicitly mentioned the SDGs as a motivating factor. Second, the authors argue that in response to various ‘reality checks’ from academics and civil society that a bioeconomy transformation may not deliver on its promises of economic growth and may face significant environmental challenges, policymakers, particularly in the EU and other OECD countries, have shifted to a ‘green growth’ framing, in which bioeconomy growth is expected to lead to greater sustainability. Indeed, our data also support this notion by showing that environmental goals are more prominent in high-income countries than in non-high-income countries.^8^ Specifically, as shown in Appendix S6, the share of environmental goal text is 10% higher in high-income countries than in their non-high-income counterparts (49% and 39%, respectively).^9^

It should be noted, however, that many scholars remain skeptical about the sincerity of policymakers’ environmental goals. Recent studies have found that the increased salience of environmental goals has often not translated into increased resource allocation to achieving these goals (Holmgren et al. 2020; Lühmann 2020). This has led some researchers to view this increased salience primarily as a rhetorical strategy to promote or defend the bioeconomy and its economic growth promises against criticism (Ramcilovic-Suominen and Pülzl 2018; D’Amato et al. 2020; Lühmann and Vogelpohl 2023).

The finding that the *political* goals category comes in second may seem surprising at first glance. Several studies have found that state bioeconomy discourses are technocratic and background their political character (Lühmann 2020; Töller et al. 2021; Vogelpohl and Töller 2021). A closer look at the subcategories reveals that many of the goals in this category relate to technocratic governance and regulatory considerations rather than politics, participation, or ideational and power struggles.

The relative salience of the *research, innovation and technology* goals category further reflects the much-noted techno-centric nature of the bioeconomy discourse (Hausknost et al. 2017; Meyer 2017; Vivien et al. 2019; Ranacher et al. 2020; Vogelpohl and Töller 2021; Eversberg et al. 2023). Its lower salience compared to the economic goals’ category might reflect a goal hierarchy and an instrumental role of research, innovation and technology for economic objectives. Relatedly, the relatively low salience of the *social* goals category resonates with the limited treatment of social concerns in previous studies and with observations that the bioeconomy discourses and policies have not paid enough attention to issues of social inclusion (e.g., smallholder participation), societal dialogue (e.g., local councils), and social innovation (e.g., new consumption models) (Gerhardt et al. 2022).

**Fig. 4:**
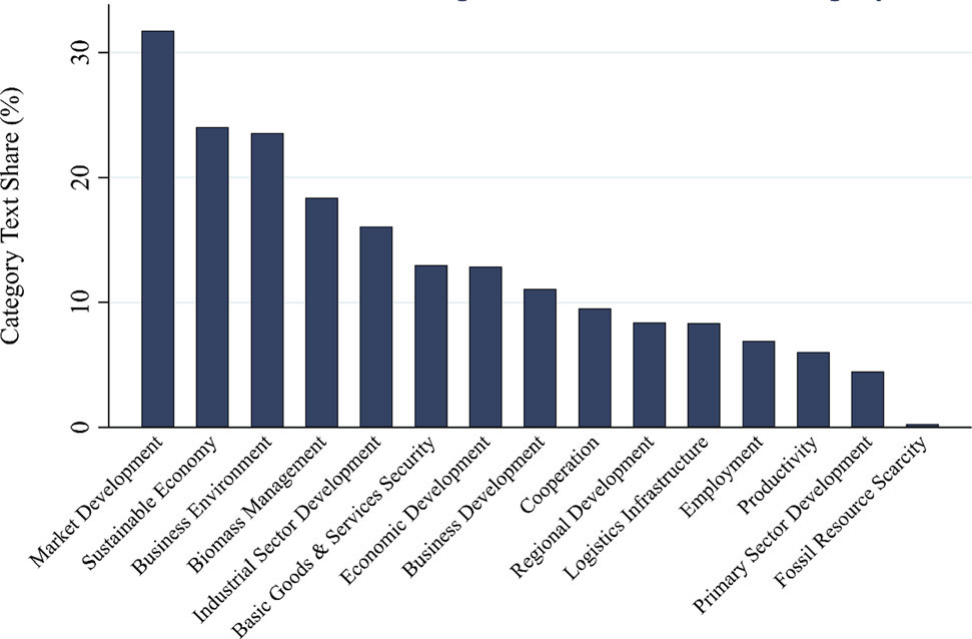
Text share of subcategories in the economic goals’ category (in percent)

To better understand which specific goals each category actually encompasses, we disaggregate them in the subsequent paragraphs and figures. First, Fig. 4 depicts the text share of the 15 subcategories within the text shares that address *economic goals,* averaged across bioeconomy documents. This disaggregation provides insights into the most prominent economic goals.

First, in line with earlier findings, the dominant orientation is the creation of bio-based products that can compete with fossil-based products in both domestic and international markets. The most salient subcategory, *enhanced market development* (31.8%), reflects a mostly market- and producer-oriented approach to the bioeconomy. In addition, the subcategories *economic development* and *growth at large*, *business development*, and *employment* indicate a strong emphasis on economic growth. A second group of subcategories such as *improved business environment* (23.6%), *cooperation along the value chain*, and *logistics and infrastructure*, points toward the need for conducive framework conditions, coordination, and cooperation. The related subcategory *development of the industrial sector* (fifth-ranked with 16.1%) has some mercantilist overtones. Third, codes related to *sustainable economy* (24.1%), the second most frequent single category of economic goals, demonstrate the influence of the sustainability discourse. They might include some links to bioecology visions. Fourth, the relative importance of the subcategories *biomass management* (18.4%), together with the subcategories *secure provision of basic goods and services* and *fossil resource scarcity*, provides preliminary evidence of the influence of bio-resource visions in terms of economic goals. Finally, traditional goals of rural, agricultural and forestry policy, such as *regional development* and *primary sector development*, are not particularly salient in our text corpus.

**Fig. 5:**
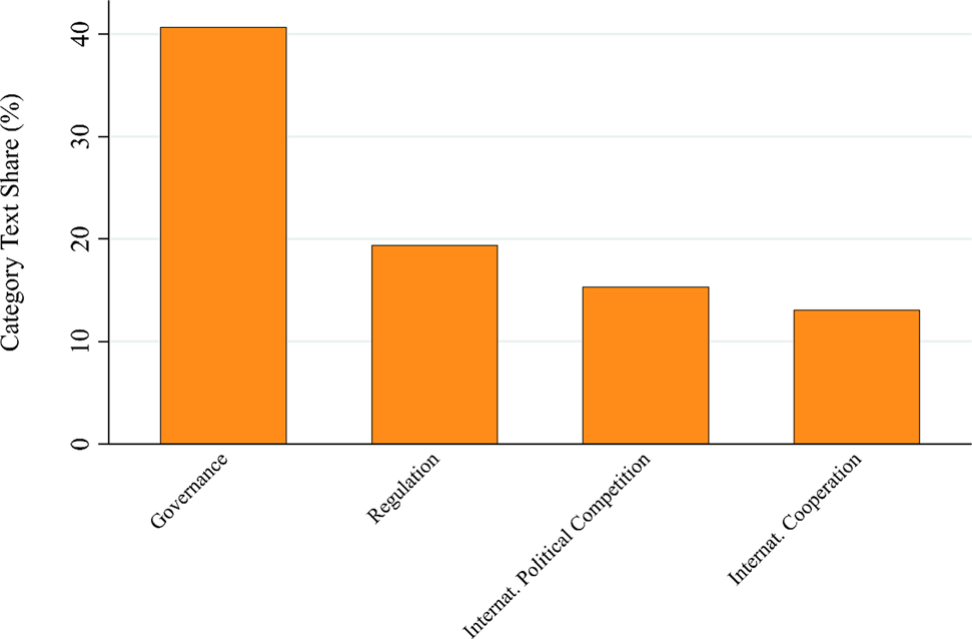
Text share of subcategories in the political goals’ category (in percent)

Next, we take a closer look at the *political goals*’ category. The stated goals in this dimension are less differentiated with four major subcategories: governance, regulation, international political competition, and international cooperation (see Fig. 5). Codes in the subcategory *governance* were by far the most frequent, accounting for 40.7% of the category’s text share. Further disaggregation of this subcategory reveals a dominantly technocratic focus (see Appendix S7b). Specific stated aims are, inter alia, to increase the strategic capacity of the state to monitor and support the bioeconomy and its sub-sectors, to promote inter-agency coordination, and to create an institutional environment conducive to the development of the bioeconomy. Relatedly, the subcategory *regulation* (which accounts for 19.4% of the text share) mainly captures the desire to create regulatory frameworks and laws that accommodate the emerging requirements of the bioeconomy. While the category *international cooperation* (13.1%) mainly addresses questions of international harmonization and coordination, the category *international political competition* (15.4%) is primarily concerned with avoiding dependence on foreign supplies.

**Fig. 6:**
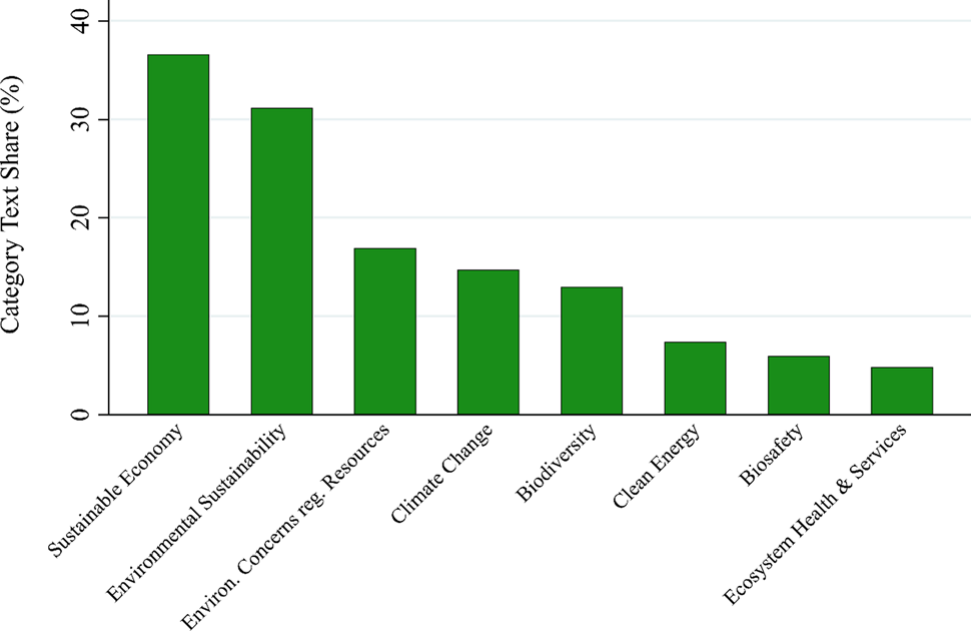
Text share of subcategories in the environmental goals’ category (in percent)

Within the *environmental goals*’ category, the most salient subcategories are *sustainable economy* (36.6%) and *environmental sustainability* (31.2%), as shown in Fig. 6. Both relate environmental goals to the development of the economy, indicating that the dominantly economic perspective of the bioeconomy visions in our text corpus is also reflected in the formulation of environmental goals. The latter is mostly driven by one code, *sustainable resource management* (25.9%). Within the *sustainable economy* subcategory, the most frequent codes are *sustainable economy and clean growth* (12.8%) and *circular economy* (7.9%) (see Appendix S7c). Four subcategories are concerned with environmental effects of the bioeconomy: *environmental concerns regarding resources and bio-based production systems* (16.9%), *clean energy* (7.4%), *biosafety* (5.9%), and *ecosystem health and services* (4.8%). Two subcategories address general environmental issue areas: *climate change* (11.6%, mostly driven by *climate change mitigation* with 11.2%), and *biodiversity* (13%).^10^

**Fig. 7:**
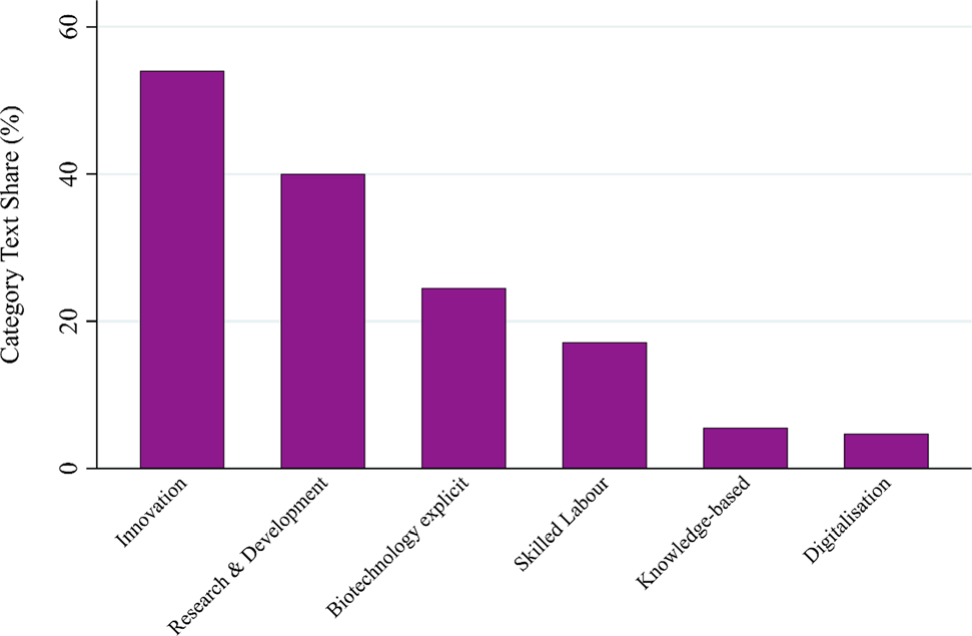
Text share of subcategories in the research, innovation & technology goals category (in percent)

In the category *research, innovation & technology goals* (see Fig. 7), the two most salient subcategories are *innovation* (54%) and *research & development* (40%), followed by the more narrow subcategories *biotechnology* (24.5%) and *skilled labor* (17.2%), *knowledge-based* (5.5%) and *digitalization* (4.7%). First, the strong salience of the first two subcategories supports previous findings that innovation, research and technology development are key objectives of most bioeconomy-related strategies (D‘Amato et al. 2017; Meyer 2017; Vivien et al. 2019; Böcher et al. 2020). However, the codes under these subcategories often refer to quite generic statements, and it is often not clear whether they support a more bioresource or biotechnology-oriented vision (see Appendix S7d). Indeed, the lower salience of the subcategory *biotechnology* within the category provides some indication that the biotechnology vision may not be globally dominant (Aguilar et al. 2013; Meyer 2017). Second, our results show that the discourse of the “knowledge-based bioeconomy,” historically particularly important in the European Union in the 2000s (Hausknost et al. 2017), has left traces in our text corpus. Finally, the digitalization discourse, which has played a major role in recent academic and policy debates on the bioeconomy (Klitkou et al. 2017; Watanabe et al. 2019; OECD 2020; Asveld 2021), has also found its way into the objectives of bioeconomy policies in our text corpus.

**Fig. 8:**
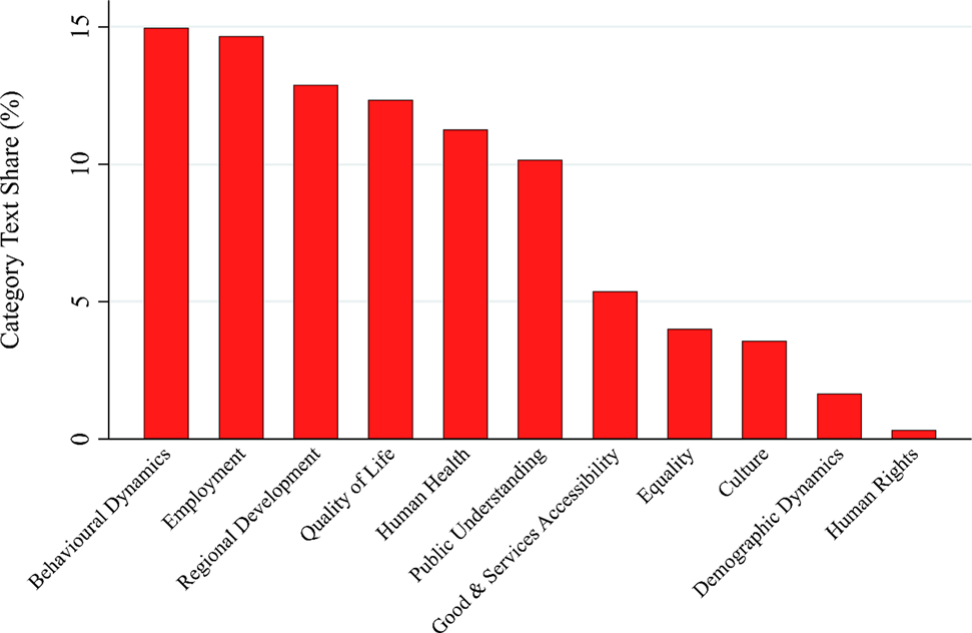
Text share of subcategories in the social goals’ category (in percent)

The *social goals* category contains a range of different types of objectives, none of which has an outstanding salience – the most salient subcategory, *behavioral dynamics*, appears in 15% of the text share that contains social objectives (see Fig. 8). Key codes within this category are *consumer behavior*, *producer behavior*, *social acceptance of bio-based products*, and *ethical concerns* (see also Appendix S7e). The three most salient subcategories are related to economic issues: *Behavioral dynamics* is mostly concerned with the acceptance of bio-based production systems and products and is thus closely related to market development (which is also partially true for the category “public understanding”). *Employment* (14.7%) is also essentially a key economic consideration, as is *regional development* (12.9%). This finding confirms the dominance of economic objectives in our text corpus. The subcategory *public understanding* (10.2%) is often linked to the objective of acceptance of bioeconomy and biotechnology. However, the text corpus also contains a range of genuinely social objectives, which were summarized under the subcategories: *quality of life* (12.3%), *human health* (11.3%), *goods and services accessibility* (5.4%), *equality* (4%), *culture* (3.6%), *demographic dynamics* (1.7%), and *human rights* (0.3%).

Finally, it is worth briefly discussing the role that global equity could play in bioeconomy strategies. To date, this cross-cutting issue has received considerable attention from social scientists and civil society organizations worldwide (for a comprehensive overview, see Backhouse et al. 2021a). Specific areas of concern include unequal trade relations, where high-income countries often source much of their biomass needs from low-income countries, leading to land use conflicts and land conversion in producing countries. We find that such issues are not strongly reflected in the sampled policy documents. The goals of reducing geopolitical and global inequalities account for only 0.14 and 0.04% of the average document’s goal text, respectively. Interestingly, both goals are more often mentioned by high-income countries. The picture is slightly different for trade relations. On average, a less trivial 0.8% of the text is devoted to creating a more level playing field in trade. Here, the text share is higher in non-high-income countries (1%) than in high-income countries (0.6%), probably reflecting a greater awareness of such inequalities. This is also in line with the figures presented earlier for the goal of supply independence from other countries. Non-high-income countries mention this as an objective 3% of the time, twice as many as high-income countries (1.4%). However, on closer examination of the underlying textual statements, these percentages begin to reflect more classic center-periphery relations. Specifically, while high-income countries (e.g., France or the United States of America) seek to become less dependent on imported raw materials (both biological and fossil), non-high-income (e.g., Kenya, Malaysia, or South Africa) countries seek to replace consumer goods, such as biomedicines, with domestic production. Importantly, the previously discussed goal code of land competition (including issues such as land use conflicts and land conversion) is more prominent in non-high-income countries (1.6%) than in high-income countries (0.5%). This is consistent with the argument in the literature that such issues are of particular concern in lower-income countries. However, it also suggests that wealthier countries do not want to problematize or prioritize such issues existing outside of their countries in their strategies.

### The relative importance of bioeconomy visions

**Fig. 9:**
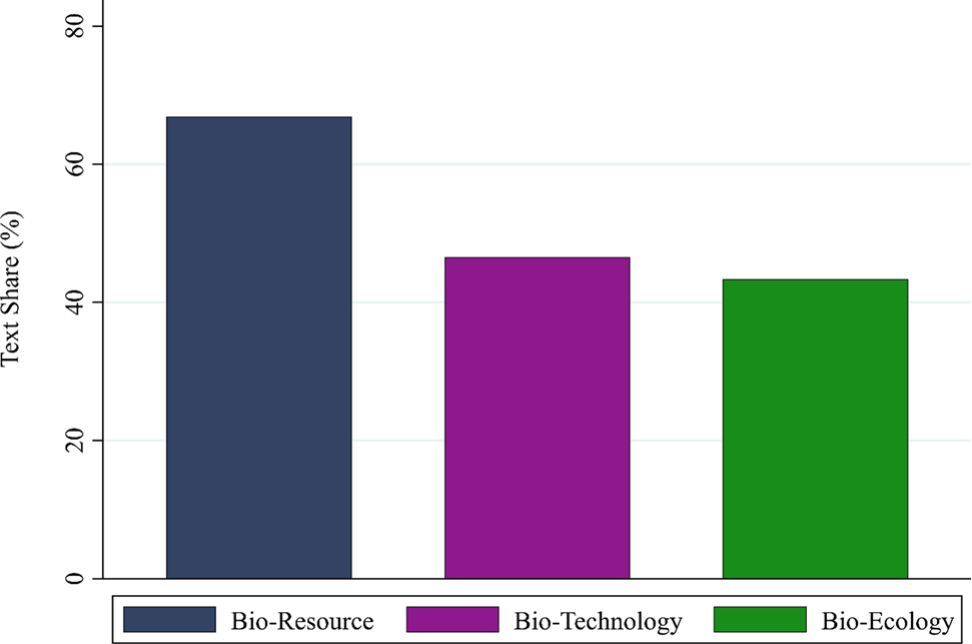
Share of vision types in goal-related text share of all bioeconomy-related policy documents (in percent) (Our coding did not distinguish between biodiversity as an area worth protecting and one that can be utilized for the bioeconomy)

In the next step, we analyze the orientation of visions in the bioeconomy-related policies in our sample. For this purpose, we assess the salience of objectives which can be linked to the different vision categories. Figure 9 shows the average share of text containing objectives linked to each of the vision types within those text shares in which any goal was coded (i.e., text shares that did not contain any goal statements are excluded from the denominator). Several results emerge. The bioresource vision is by far the most salient vision type. Goals related to this vision were found in nearly 67% of all goal-related text. The most salient codes related to the bioresource vision are *research and development*, *biomass management*, and *economic development/growth* (see Appendix S8a). The other two vision types have a similar but lower salience. The codes *research and development*, *biotechnology explicit*, and *economic development/growt*h are the most salient codes linked to the biotechnology vision (see Appendix S8b), while the codes *environmental sustainability*, *environmental concerns*, and *sustainable economy/clean growth* are characteristics of the bioecology vision (see Appendix S8c). As shown in Appendix S8, similar results emerge when assessing the extent to which different vision categories are represented only within bioeconomy strategies.

These findings show the bioresource vision as the most salient one in our text sample and the bioecology vision as nearly as salient as the biotechnology vision. This is somehow contrary to earlier assessments which found the biotechnology and bioresource visions as equally important and the bioecology vision less influential in governmental policies. Importantly, however, it should be noted that some of the codes linked to the bioecology vision in our analysis, especially the *sustainable economy* codes, may not be fully aligned with its original intent. For example, while the bioecology vision entails a strong sustainability norm (Neumayer 2004), some of the goals coded under *environmental sustainability* might rather amount to weak sustainability. It is also important to highlight that the bioecology vision inherently prioritizes environmental concerns over economic goals. However, of our 78 documents, only four show codes assigned to the bioecology vision that outweigh those assigned to the bioresource or biotechnology visions in terms of text share.^11^ Moreover, many of the goals coded for the bioresource and biotechnology visions, such as increasing economic growth through increased industrial productivity or the use of genetic modification, are inconsistent with the more sufficiency, ecology, and degrowth-oriented aspirations of the bioecology vision. Consequently, while there are a significant number of goals that are superficially consistent with the bioecological vision, the majority are not. As a result, we do not consider any of the strategies to be broadly consistent with, or in line with, the bioecology vision. As noted above, it remains questionable to what extent policymakers will actually act on the more ecologically and environmentally framed goals.

**Fig. 10:**
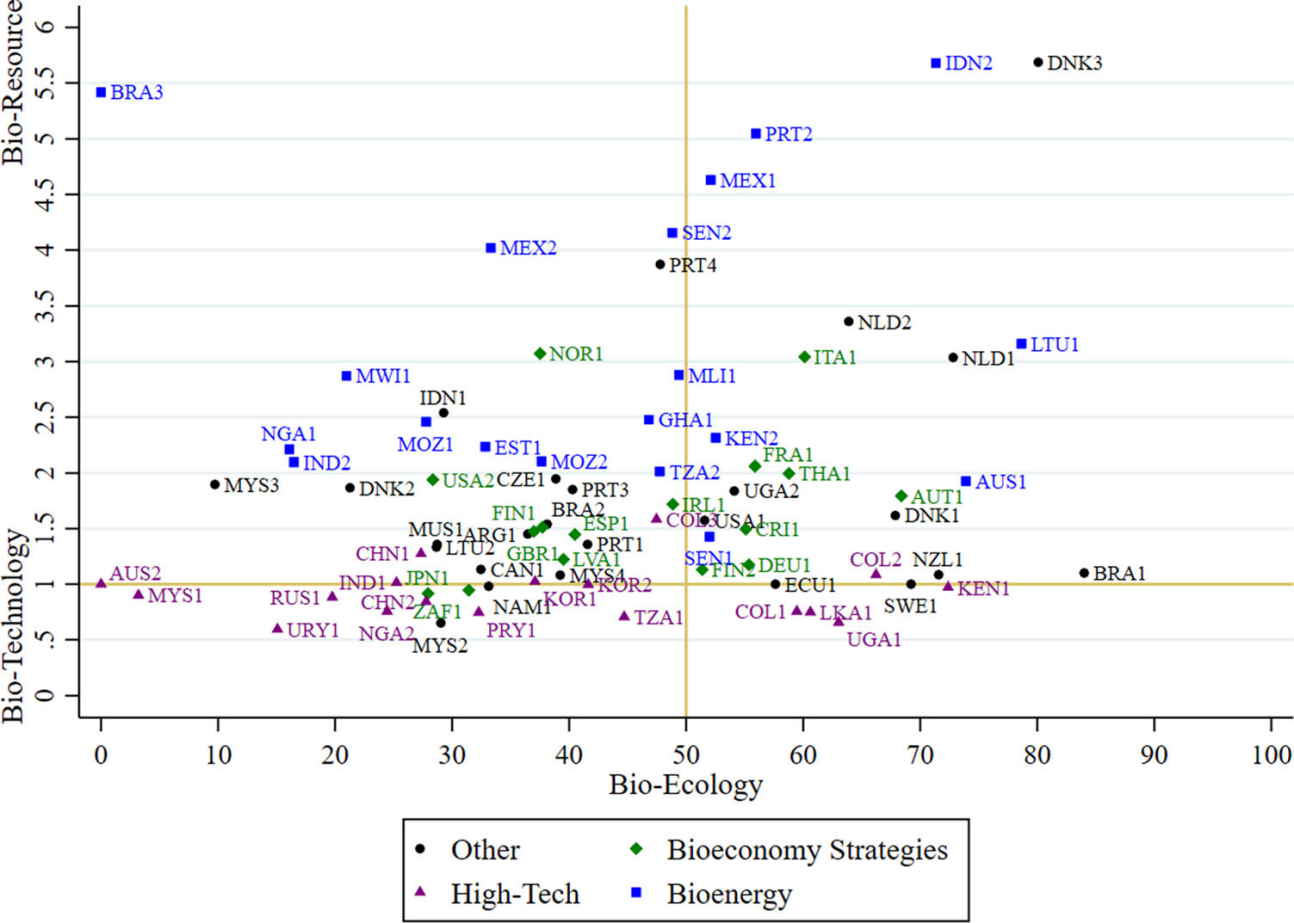
Scattering of countries and document types within bioeconomy visions (These are Colombia’s 2016 ‘Colombia Bio’ strategy, Tanzania’s 2014 ‘Biomass Energy Strategy,’ Ecuador’s 2019 Bioentrepreneurship guidelines, and Portuals 2013 ‘National Ocean Strategy.’)

In the third step of the analysis, we differentiate the results by country. The scatterplot in Fig. 10 provides a more comprehensive representation of bioeconomy visions for each country and document type along two axes. The horizontal axis shows the proportion of goal-related text in each document coded with bioecology-oriented codes. The vertical axis relates the respective text shares of the bioresource and bio-technology visions. Thus, a score of 2 on the vertical axis would imply that a document’s share of bioresource-related text is twice as large as its share of biotechnology-related text. A score of 0.5 would indicate the opposite. A score of 1 indicates equal emphasis. We categorize our sample according to the three most frequent document types (bioeconomy strategies, high-tech and bioenergy policies, see also Appendix S12) and aggregate all remaining documents as “other.”

Overall, the majority of documents has less than 50% text share with bioecology-oriented goals, which is consistent with our previous findings. Bioeconomy strategies do not deviate from this pattern. They mostly cluster around the center of the graph, which can be explained by their encompassing nature. Specifically, they have average levels of bioecological orientation and tend to be slightly more bioresource than biotechnology-oriented. Moreover, the finding that there are no clear outliers in this group suggests that bioeconomy strategies tend to present balanced, integrated, or hybrid visions that combine elements of the biotechnology, bioresources, and bioecological visions.

Biotechnology documents, which mostly focus on high-tech approaches to the bioeconomy, mostly fall below the horizontal 1-point line, indicating a dominant orientation toward the biotechnological vision, suggesting that our coding is valid in this regard. 72% of high-tech bioeconomy policies score lower on bioecological vision than the average bioecological score of all other document types (45%). This suggests that these biotechnology strategies tend to be relatively less ecologically oriented.

All bioenergy-focused documents in our sample score high on the bioresource vision, while they spread widely on the bioecology vision. The first observation is consistent with the increased production of biomass for bioenergy (and especially biogas) purposes (IEA Bioenergy 2021), the second requires further analysis of the underlying factors that could explain this strong variation.

At the level of individual countries, different documents from the same country do not necessarily form clusters of similar vision profiles. An example of a relatively coherent pattern is Malaysia, where all four documents are found on the left side of the bioecology dimension, indicating values below-average. Still, the values in the biotechnology and bioresources dimension show significant variation and land on both sides of the median. Extremely divergent vision profiles are found for Brazil, whose 2016 policy “Estrategia Nacional de Ciencia, Technologia e Inovacao” (BRA1) has the highest bioecology text share in the entire text corpus, while the country’s 2020 policy “Plano Decenal de Expansão de Energia 2029” (BRA3) has the lowest text share regarding bioecology. In contrast, Australia’s 2014 bioenergy policy “Opportunities for Primary Industries in the Bioenergy Sector” (AUS1) is more bioecologically oriented than the country’s “Biotechnology and agriculture in Australia” policy (AUS2), which has an extremely low value for its bioecological orientation. Whether these differences reflect shifting policy orientations over time or simply different substantive foci of the strategies is a question for future research.

## Conclusion

Increased utilization, management, and exploitation of biological processes and renewable resources are widely expected to become an important area of economic growth. The wide range of activities in this field, summarized under the term bioeconomy, comprises very different actors, technologies, and markets and cannot be expected to form a coherent entity. Consequently, the emerging bioeconomy, positioned at the interface of agricultural and industrial activities and with complex links to land use and resource supply, has stimulated very different expectations and concerns. These have been consolidated into distinguishable and competing bioeconomy visions which emphasize either the use of advanced biotechnologies, the importance of bioresources or the ecological embeddedness of the bioeconomy (Bugge et al. 2016; Vivien et al. 2019).

The adoption of these bioeconomy visions in governmental policy documents is an important step toward their institutionalization. Governmental bioeconomy strategies provide recognition and legitimacy and guide policy initiatives. It is therefore important to understand which bioeconomy visions prevail in these strategies. For this purpose, our qualitative content analysis of 78 bioeconomy policy documents from 50 countries identified stated policy goals and grouped them into overarching categories which were then linked to the main bioeconomy visions. The underlying assumption is that the salience of stated goals is a valid indicator for predominant governmental bioeconomy visions.

In the analyzed bioeconomy strategies, economic goals predominate and are mainly related to market development, sustainable economy, and biomass management. Environmental goals are dominated by objectives related to a sustainable economy and sustainable resource management, reflecting a predominantly economic perspective even in the articulation of environmental objectives. Importantly, the gap between the respective text shares of economic and environmental goals has narrowed since 2015. This seems to reflect a trend toward framing bioeconomy strategies more in terms of sustainability and green growth, especially in high-income countries. Political goals are mostly related to technocratic governance and regulation. Similarly, stated social goals are mostly related to economic issues or technology acceptance, while genuinely social objectives such as quality of life, human health, and addressing inequalities are less salient. Goals related to research, innovation and technology in general were more salient than specific biotechnology-related objectives, indicating a less dominant role of the biotechnology vision than diagnosed in earlier research (O’Mahony 1999; Leitch and Motion 2009). Concerns about global justice, while much discussed among social scientists studying the bioeconomy, are not strongly reflected in bioeconomy policy documents.

In line with earlier studies looking at the salience of bioeconomy visions in European bioeconomy policies and bioeconomy research more generally (Meyer 2017; D’Amato et al. 2020), our encompassing sample showed the bioresource vision as most salient. Goals related to bioecological visions are more salient than expected from earlier assessments (Hausknost et al. 2017; Scordato et al. 2017; Tittor 2021; Vogelpohl and Töller 2021), being more present in bioenergy policies and bioeconomy strategies than in biotechnology-focused policies. It should, however, be clearly noted that we do not consider any of the sampled documents to have a bioecological vision, given their overall strong economic focus and alignment to a weak understanding of sustainability. Finally, documents from the same country do not necessarily show the same vision type orientation**.**

The results presented in this paper are limited to the analysis of stated goals in governmental text documents. Further analysis should include other layers of policy formulation, such as instruments, but also problematizations and stated rationales. To develop a critical perspective on such documents, in-depth case studies are needed to understand how alternative ideas and interests were excluded or backgrounded, and why several strategies with different bioeconomy visions are published in one country. It would also be fruitful to extend the analysis to include bioeconomy-related strategies and statements by non-state actors. These tend to help qualify government strategies and goals, while representing important views of often-marginalized interest groups. Further research should also assess the impact of governmental bioeconomy strategies on policymaking, governmental programs, and regulatory initiatives, e.g., through process tracing. Long-term studies are needed to understand shifts in the bioeconomy discourse and the prevalence of competing bioeconomy visions, including the possible emergence of new visions. This includes the articulation of ‘hybrid’ visions that combine elements of several competing visions. Complemented by the findings of this study, such future research has the potential to significantly deepen our understanding of the nature and evolution of bioeconomy politics, policy, and discourse.

### Supplementary Information

Below is the link to the electronic supplementary material.

Supplementary file1 (PDF 891 KB)

## Footnotes

1 The food-energy dilemma refers to the food vs. fuels debate associated with (in)direct land use changes due the increased production of energy crops (e.g., maize) especially for the generation of biogas. Moreover, the environment–development nexus is linked to the long-standing sustainability growth debate and the discussion about the reconciliation of environmental and economic sustainability. Finally, the society technologization challenge goes along with biosafety (e.g. human health), biodiversity, labor market, and ethical issues induced by the increased use of (bio)technology.

2 To identify potential policy documents, we conducted a Google search using the following search terms: "(policy | strategy | plan | política | estrategia | politique | stratégie) and (bioeconomy | bio-economy | biotechnology | biomass | biofuel | bioenergy | bioindustry | circular | bioeconomía | biotecnología | biomasa | biocombustible | bioenergía | bioéconomie | biotechnologie | biomasse | biocarburant | bioénergie | circulair)". We then reviewed the first 200 results, looking for direct references to national governmental bioeconomy or bioeconomy-related strategies, i.e., strategies promulgated by a national government, ministry, or ministry-linked agency. Strategies authored by sub-national governments (e.g., the regions of Flanders in Belgium or Baden-Württemberg in Germany) or non-governmental actors were excluded.

3 It should be mentioned that we are interested in a verbatim reading and coding of policy goals while an analysis of implied or hidden aims and objectives is beyond the scope of this paper. This means that we analyze the policy goals defined as the stated aims pursued by a policy (Feindt et al. 2020a, 641).

4 To ensure intercoder reliability, the first document was coded jointly by all three coders, the second and third documents were coded in parallel by all coders and then compared and discussed using MAXQDA's intercoder agreement functions, and a final round of intercoder reliability checks using the same functions was conducted after each coder's tenth coded document. Any potential coding ambiguities were immediately discussed with the entire team throughout the coding process.

5 In rare instances, codes and subcategories could be assigned to more than one category. For example, the subcategory "employment" was assigned to both the "economic" and "social" categories.

6 Note that we assigned some keyword-matching codes to certain vision types only when coded alongside specific scope conditions (e.g., sectors or products which we coded as well), as otherwise they might be too broad. A key example is our category "new products/processes/technologies," which was only assigned to our vision type " biotechnology" when coded alongside specific sectors and products, such as "manufacture of basic pharmaceutical products" or "manufacture of chemicals and chemical products." These cases are shown in Appendix S5. Note also that we created a narrower version of each vision. Included codes are italicized in Appendix S5. The use of this is twofold. One, for the bioresources and biotechnology visions there are a couple of more generic codes, such as “economic growth,” which are so frequently coded and assigned to both visions, that it can sometimes be difficult to distinguish how prominent more vision specific coding (e.g. ‘biomass management’ or ‘bioprospecting) is. A narrower version here helps readers to identify the relative importance of these two visions. The narrower version of the bioecology vision excludes a couple of codes that are more ambiguous and whether they should be considered as part of that vision, because of their more economic nature, such as ‘sustainable economy’ or ‘regional development/growth.’.

7 Note: Because a text segment can be coded with different codes belonging to different subcategories and categories, and because some codes are assigned to several distinct categories and types, the combined percentage of such derived variables may exceed 100%.

8 We use the World Bank’s country classification by income group to make this distinction.

9 Other than that, the relative shares across the categories are broadly consistent. It should also be noted that there might be other factors that collectively lead governments of high-income countries to place greater emphasis on environmental goals, such as: greater resource availability, enhanced regulatory capacity, higher levels of public awareness and education, a less environmentally damaging economic structure, and greater international pressure to meet environmental commitments.

10 Our coding did not distinguish between biodiversity as an area worth protecting and one that can be utilized for the bioeconomy.

11 These are Colombia’s 2016 ‘Colombia Bio’ strategy, Tanzania’s 2014 ‘Biomass Energy Strategy,’ Ecuador’s 2019 Bioentrepreneurship guidelines, and Portuals 2013 ‘National Ocean Strategy.’
