# Supplementary material for: A global analysis of bioeconomy visions in governmental bioeconomy strategies
Source: Ambio. 2023 Dec 27;53(3):376–88. doi: 10.1007/s13280-023-01958-6 (PMC10837399; doi:10.1007/s13280-023-01958-6)
Supplement: Supplementary file 1 — Supplementary file1 (PDF 891 KB) [file 13280_2023_1958_MOESM1_ESM.pdf]

Ambio

Supplementary Information
This supplementary information has not been peer reviewed.

Title: **A Global Analysis of Bioeconomy Visions in Governmental Bioeconomy Strategies**

Authors: Maria Proestou, Nicolai Schulz, Peter H. Feindt

Content

Appendix S1: Number of documents per document type (as per GBS 2020 report perspective) ... 3
Appendix S2: Number of documents per year ... 4
Appendix S3: Literature-based keyword per vision type ... 5
Appendix S4: Topic coding scheme ... 7
Appendix S5: Allocation of codes to bioeconomy visions ... 20
Appendix S6: Text share of goal categories across income groups (in percent) ... 22
Appendix S7a: 20 most frequent Economic goal codes by sub-category ... 23
Appendix S7b: Frequency of Political goal codes by sub-categories ... 24
Appendix S7c: 20 most frequent Environmental goal codes by sub-category ... 25
Appendix S7d: Frequency of Research, Innovation and Technology codes by sub-categories ... 26
Appendix S7e: 20 most frequent Social goal codes by sub-category ... 27
Appendix S8a: The relative salience of codes making up the Bio-Resource Vision category ... 28
Appendix S8b: The relative salience of codes making up the Bio-Technology Vision category ... 29
Appendix S8c: The relative salience of codes making up the Bio-Ecology Vision category ... 30
Appendix S9: Share of vision types in goal-related text share of bioeconomy strategies (in percent) ... 31
Appendix S10: Share of vision types in goal-related text share compared between the wide and narrow coding frameworks (in percent) ... 32
Appendix S11: Scattering of countries and document types within bioeconomy visions for the narrow coding framework ... 33
Appendix S12: Salience of visions and goal categories by document types and country (text shares in % of goal-coded text) ... 34

Appendix S1: Number of documents per document type (as per GBS 2020 report perspective)

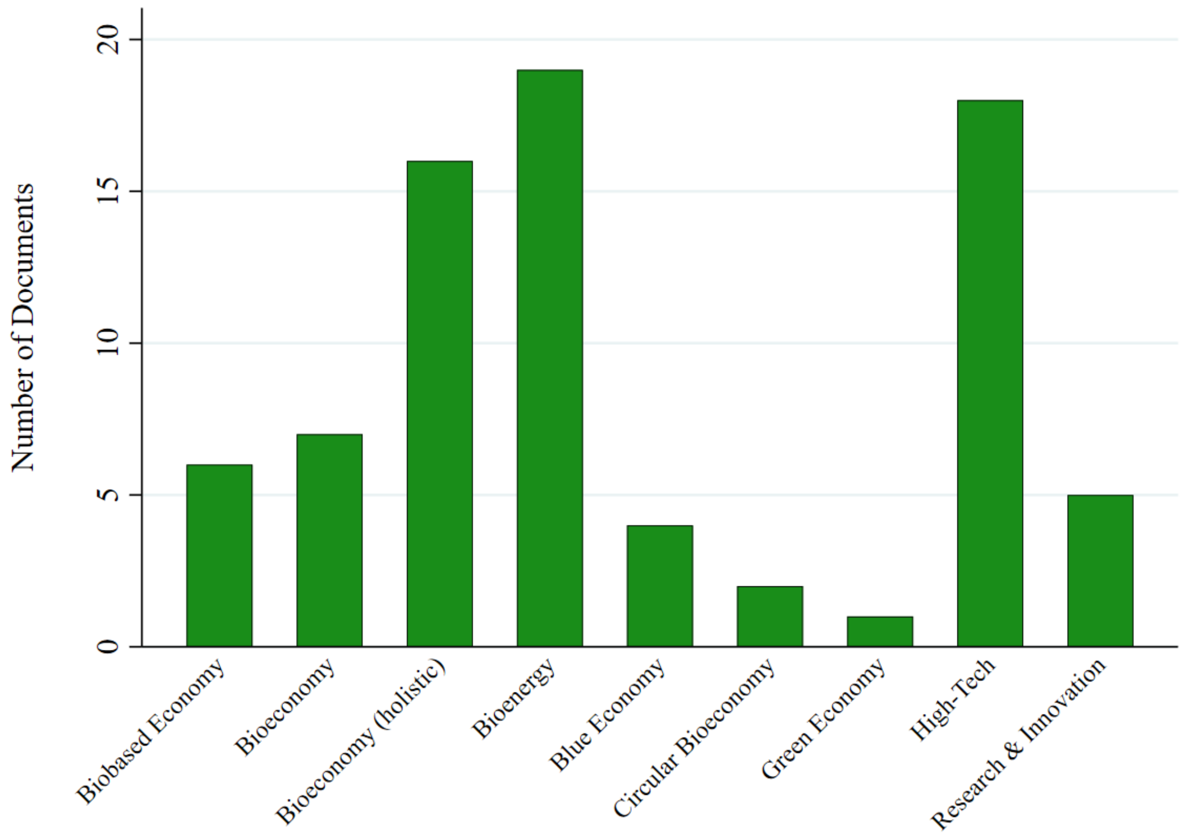

Note: "Bioeconomy (holistic)" is identical to what we term a (dedicated) bioeconomy strategy in the paper.

Appendix S2: Number of documents per year

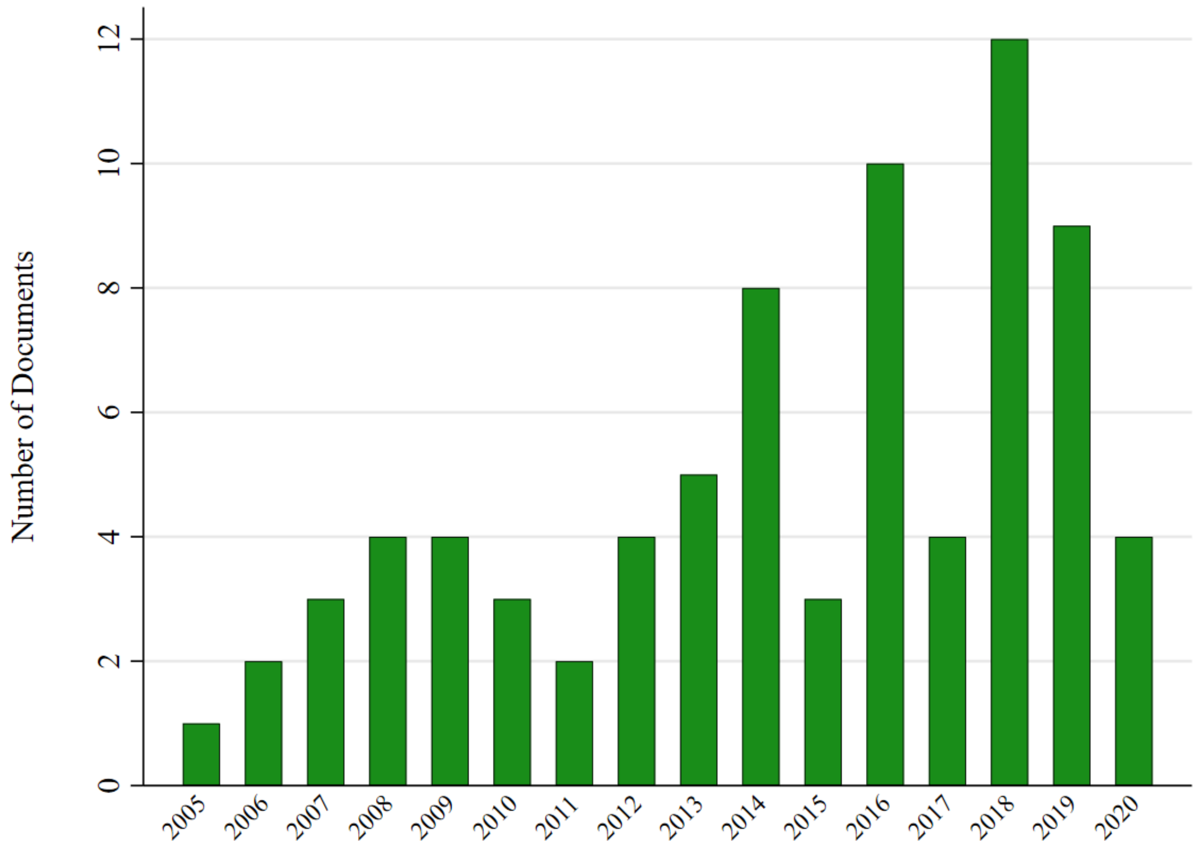

Appendix S3: Literature-based keyword per vision type

**Bio-Technology**	**Bio-Resource**	**Bio-Ecology**
Biotechnology<br>Economic growth<br>Job creation<br>Commercialization of research & technology<br>R & D, patents, TTOs<br>Research councils and funders (science push, linear model)<br>Innovations<br>Genetic engineering<br>Knowledge from life science<br>Capital-intensive knowledge production<br>Privatizable knowledge<br>International competitiveness<br>Globalized economy<br>Limited number of leading innovation regions<br>Global clusters/ Central regions<br>Genetically modified crops<br>Industrial products, biofuels, health products<br>Biological research and emerging technologies<br>More 'disruptive and radical innovations' compared to the resource-oriented bioeconomy<br>Health applications, such as personalized medicine, biopharmaceuticals, and biocosmetics; increasing crop productivity by means of engineered diversification or improvement (i.e. genetic modification); biopesticides, biofertilizers, bio-stimulants, and bio-based chemicals<br>Commercialization of research and technology	Interdisciplinary, process-oriented development of technologies for value chains<br>Scientific knowledge for intelligent production systems<br>Public–private partnerships<br>Capitalising on bio-resources<br>Conversion of biomass into new products via new value chains<br>Minimizing organic waste production and cascading use<br>International competitiveness<br>Economic growth<br>National to global value chains<br>Rural regions development<br>Linking agriculture with industrial and energy production<br>Sustainable intensification with higher yields, more efficient input use; Science- and data-based (external) management systems<br>Industrial products, bioenergy<br>Sustainability certification<br>More efficient use of biomass resources via new conversion technologies and multiple products<br>Interdisciplinary, optimization of land use, include degraded land in the production of biofuels, use and availability of bio-resources, waste management, engineering, science & market (Interactive & networked production mode) & sustainability<br>Rural/Peripheral regions<br>The main focus is on technological development of new bio-based products, and much less on environmental protection<br>In terms of value creation, the bio-resource vision highlights the processing and conversion of bio-resources into new products. Related to the use and	Agroecological techniques and methods for sustainable use of scarce natural resources<br>Farmers' collective, experimental knowledge; Scientific knowledge on agroecology<br>Capitalizing on ecosystems<br>Emphasis on quality of food and food culture<br>Provision of public goods such as biodiversity, landscapes, rural development<br>Re-localizing agro-food-energy production and consumption<br>Place-based local/regional networks of value chains; Territorial identities—terroir<br>Agroecological production with closed loop nutrient and energy cycles, enhanced soil fertility, high diversity and biocontrol agents; Minimizing external inputs<br>Food products (quality)<br>Comprehensive qualities of biomass: characterized by cultivation methods, cultural value and/or territorial identity;<br>Food certification schemes (e.g., organic certification)<br>Reduced biomass resource demand via circular economy and social innovation (e.g., sustainable consumption)<br>Sustainability, biodiversity, conservation of ecosystems, avoiding soil degradation<br>Development of integrated production systems and high-quality products with territorial identity<br>Identification of favorable organic agro-ecological practices, ethics, risk, transdisciplinary sustainability, ecological interactions, re-use & recycling of waste, land use, (Circular and self-sustained production mode)<br>Rural/Peripheral regions<br>Socio-ecological resilience<br>Diversified crops

A concentration of growth in a limited number of regions globally that host a combination of large pharmaceutical firms, small biotech firms, and venture capital<br>Interaction between universities and industry is needed	availability of bio-resources, waste management also takes up a more prominent position in the bio-resource vision.<br>Renewable energy production<br>The concept of cascading use of biomass is central in this regard since it highlights the efforts to maximize the efficiency of biomass use. Finally, it is also argued that processing of waste that allows recycling by converting it to fertilizers is central to allow large-scale biofuel production<br>The issue of land use constitutes a more explicit element than in the bio-technology vision<br>Biomass replaces fossil fuels and mining to produce energy and materials<br>Biorefining at the heart of ecological transition (multilevel perspective).<br>Identification and stakeholder coordination Substitution of products or functions by new product<br>Improve land productivity and to include degraded land in the production of biofuels<br>Availability of bio-resources is prominent<br>Role of research and innovation activities as an important driver for value creation. However, while the former takes a narrower point of departure in bio-technology research, the latter emphasizes the importance of research in multiple fields, which are in different ways related to biological materials. Consequently, research and innovation efforts often involve collaboration between actors with dissimilar competences, and the importance of research on issues such as consumer preferences is also stressed.<br>Innovation is also understood to require collaboration across sectors, e.g., that firms from the forestry industry engage closely with downstream actors.<br>Aim of ensuring transition to an economically viable use of biomass.	The use of own waste as well as waste from urban areas<br>Food security, diversity, and sovereignty are central elements in this vision, especially in emerging economies<br>The aims and objectives of the bio-ecology vision are primarily concerned with sustainability. While economic growth and employment creation is a main concern in the bio-technology and bio-resource visions, these aspects are clearly secondary to sustainability concerns in the bio-ecology vision<br>Examples of topics that are discussed are the ethics of commercialization of bioresources, safety in blood supply, inequalities in access to bio-resources, or moral dilemmas of surrogacy.<br>Biodiversity, conservation of ecosystems, the ability to provide ecosystem services, and prevention of soil degradation<br>Reuse and recycling. own waste<br>Circular and self-sustained<br>Organic bio-ecological<br>Call for greater emphasis in research on transdisciplinary sustainability topics<br>Cultivation potentials of sustainable biomass, global fair trade, and wider participation in discussions and decisions on transition processes.<br>Rural and peripheral regions<br>Identity<br>Locally embedded economies, i.e., “place-based agro-ecological systems”

Appendix S4: Topic coding scheme

Bio-technology Bio-Resource Bio-Ecology

Code System	Frequency
Economic	10550
Sustainable Economy	0
Circular Economy	479
Recycling	195
Sustainable economy/Clean Growth	426
Blue Economy	71
Green Economy	117
Bioeconomic Transformation/Growth (generic)	493
Economic Development/Growth (broadly speaking)	771
Primary Sector Development	157
Agricultural Development	144
Forest Economy	13
Industrial Sector Development	737
Biorefineries	162
Aquatic	1
Agroprocessing	25
Regional Development	399

Small forest owners	6
Small-holder-farmer participation/inclusion / farm diversity	33
Coastal Development	18
Regional Development/Growth	100
Urban Development	30
Rural Development	212
Basic Goods & Services Security	693
Cyber Security	4
Water Security	61
Energy security	277
Food security	349
Productivity	384
Industrial Productivity	13
Forestry Productivity	11
Agricultural Productivity	86
Plant Health	47
Animal-Related Concerns	19
Animal Health	53
Market Development	2071
Market failures	136

Asymmetric Information	20
Monopoly/market power/First Movement Advantages	11
Non-excludability/appropriability leading to investment	1
Informational externalities.	7
Coordination failure	23
Dynamic scale economies and knowledge spillovers	49
Economic Externalities	13
Commercialisation	428
BBP demand (un)certainty	40
BBP demand opportunity	91
Social Acceptance of bio-based products (1)	112
Consumer Behavior (1)	201
Producer behavior (1)	70
Domestic Market Access	36
Competitiveness	499
Bio-Bio competition	1
Biomass Production Profitability/Competitiveness	99
Cost Competition between Bio-based Products	24
Bio-Non-Bio Competition	52
Competition between Bio- and Non-Bio Renewable Energies	22

Competitive Bioeconomy VERSUS Fossil-Fuel-Based Economy (generi	72
Cost Competition between Bio- and Fossil-fuel-based Products	66
International Competitive Bioeconomy	161
Domestic Market Creation/Development	189
Foreign (direct) investment	51
Export Success/Growth/Leadership/Development	148
Foreign Market Access	70
Biomass Management	1165
Biomass (Allocation) Management	71
Land competition	104
Biomass Supply/Economic Opportunity	601
Biomass Supply (un-)certainty	189
Supply (In-)Dependence on Other Countries	137
Resource Scarcity	63
Logistics/Infrastructure	363
Business Development	213
Private Sector Capability	116
Anchor companies	25

Clusters	72
Business Environment	1055
New Businesses	314
Biotechnology firms	49
Financial Capacity/Budget	151
Incentives	298
Business climate (generic statement)	162
Financial Risk	81
Employment	366
High-Value Jobs	19
Cooperation	401
Cooperation of companies in the same value chain or util	73
Cooperation of companies across different value chains	99
Public-Private Cooperation	157
Research-Industry Cooperation	72
Fossil Resource Scarcity/Finity/Limit	44
Environmental	5474
Sustainable Economy (1)	0
Bioeconomic Transformation/Growth (1)	493
Green Economy (1)	117

Sustainable economy/Clean Growth (1)	426
Blue Economy (1)	71
Circular Economy (1)	284
Environmental Concerns with Resources and BBPS	915
Soil	135
Water	177
Air	30
Forests (as environmental concern explicitly)	144
Agricultural land (as environmental concern explicitly)	74
Arable Land (as environmental concern explicitly)	17
Grassland (as environmental concern explicitly)	6
Marine (as environnemental concernexplicitly)	68
Urban (as environmental concern explicitly)	7
Pollution	105
Pesticides	19
Plastic/Microplastic pollution	29
Land competition (1)	104
Environmental Sustainability	977
Sustainable resource management	782
Sustainable/Low carbon future	132

Common Pool Resources	28
Standards	35
Biosafety	134
Ecosystem health and services	216
Ecosystem functions & services	147
Ecosystem health	69
Climate Change	652
Climate Change mitigation	300
Climate Change adaptation	69
Climate threats	71
Ocean acidification	5
Sea level rise	4
Frost	3
Heat	3
Salinity	7
Dry Climate	8
Desertification	5
Wildfire	2
Droughts	10
Rainfalls/Flooding	7

Clean energy	170
Biodiversity	428
Habitat	24
Species	37
Genetic	78
Biomimicry	3
Social	2633
Demographic Dynamics	146
Population Growth	89
Ageing society	19
Crowding	2
Migration	15
Urbanization	21
Employment (1)	366
High-Value Jobs (1)	19
Culture	112
Culture/Cultural Capacity	56
Indigenous Knowledge (1)	56
Underdevelopment	3
(In)accessibility	116

Education (In)accessibility	5
Food (In)accessibility	3
Energy (In)accessibility	38
Medicines/Health System (In)accessibility	45
Water/Sanitation (In)accessibility	10
Drugs/Medical Supplies (In)accessibility	1
Behavioral Dynamics	521
Public Trust (generic)	20
Social Acceptance of BBPS and methods	57
Social Acceptance of bio-based products	112
Ethical concerns	61
Consumer Behavior	201
Producer behavior	70
Quality of life/Societal well-being	278
Poverty (Reduction)	68
Public understanding/knowledge of bioeconomy	205
Human Health	388
Childhood/infancy problems	5
Poisoning	11
Communicable diseases (Vaccines/Epidemics/pandemics)	50

Covid-19	22
Poliomyelitis	2
Malaria	5
Tuberculosis	8
HIV/AIDs	11
Non-communicable diseases	12
Iron deficiency	2
Pollution-based	10
Cancer	5
Malnutrition	6
Diabetes	3
Heart diseases	3
Metabolic Syndrome	2
Cardiovascular disease	2
Obesity	2
Arthritis	2
Equality (other subcategories?)	113
Age/Youth	4
Global	8
Urban-Rural	23

Income-Wealth	12
Gender	19
Ethnic/Racial	5
Human Rights	15
Minority Rights (other?)	9
Indigenous Rights	5
Political	3489
Regulation	467
Recognition of perverse incentives, unproductive paths & lock-in	45
State Intervention in Economy	37
National Regulatory substance	274
Urgency and Momentum for policy action	47
National Regulatory (un-)certainty	64
International Cooperation	223
International Political Competition	362
Supply (In-)Dependence on Other Countries (1)	137
Level Playing Field in Trade	37
Geopolitical (Outcome) Equality	9
Geopolitical Process Equality	6

National/Supra-Regional Reputation/Soft Power at International	164
Governance	2422
Strategic Capacity on a domestic level	85
Political (In)stability/Wars	21
State capacity for identifying, measuring, and/or monito	290
Bureaucracy and administration	39
Corruption	12
(Topic) Decentralized decision making	6
(Topic) Bottom-Up Decision Making	2
(Topic) Broad/Cross-stakeholder consultation	167
(Topic) Grassroot-stakeholder consultation	39
Inter-Authority-Coordination/Cooperation	214
International Harmonization	203
Institutions	126
Diversity Acknowledgement / cognitive pluralism	49
Governance/Regulation (Good Governance)	45
Research, Innovation & Technology	4526
Innovation	1753
New products/technologies/production techniques	1270
New Technologies	693

New Products	347
New Processes	155
Innovation capacity	483
Research & Development (multi- & inter-disciplinarity	942
Technological (Un)Certainty/Gap	99
Intellectual Property (Rights)	58
Skilled Labor	472
Educational/Training Schemes	186
Knowledge-based Bioeconomy (explicit)	116
Indigenous Knowledge	56
Digitalisation	133
Biotechnology (explicit)	953
Bioprospecting	70
General	100
Millennium Development Goals (MDGs)	10
Sustainable Development Goals (SDGs)	89

Appendix S5: Allocation of codes to bioeconomy visions

**Bio-Technology**	**Bio-Resource**	**Bio-Ecology**
Biotechnology firms<br>*Biotechnology (explicit)*<br>*Bioprospecting*<br>Economic Development/ Growth (broadly speaking)<br>Employment<br>*High-Value Jobs*<br>Commercialization<br>Cyber Security<br>Digitalization<br>*Research & Development (multi- & inter-disciplinarity)*<br>*Intellectual Property (Rights)*<br>*Knowledge-based Bioeconomy (explicit)*<br>*Innovation capacity*<br>Agricultural Productivity<br>*Plant Health*<br>*Animal-Related Concerns*<br>*Animal Health*<br>*Poliomyelitis*<br>*Malaria*<br>*Tuberculosis*<br>*HIV/AIDs*<br>*Cancer*<br>*Diabetes*<br>*Heart diseases*<br>*Metabolic Syndrome*<br>*Cardiovascular disease*<br>*Obesity*<br>*Arthritis*<br>International Competitive Bioeconomy<br>*Anchor companies*<br>*Clusters*<br>*Biomimicry*<br>*Medicines/Health System (In)accessibility*	*Cooperation of companies in the same value chain*<br>*Cooperation of companies across value chains*<br>*Circular Economy*<br>*Recycling*<br>*Sustainable economy/Clean Growth*<br>International Competitive Bioeconomy<br>Economic Development/Growth (broadly speaking)<br>*Primary Sector Development*<br>*Agricultural Development*<br>*Forest Economy*<br>*Industrial Sector Development*<br>*Biorefineries*<br>*Aquatic*<br>*Agro-processing* commercialization<br>*Coastal Development*<br>*Regional Development/Growth*<br>*Rural Development*<br>*Energy security*<br>*Water Security*<br>*Productivity*<br>*Industrial Productivity*<br>*Forestry Productivity*<br>*Agricultural Productivity*<br>Plant Health<br>Animal-Related Concerns<br>Animal Health<br>Common Pool Resources<br>Employment<br>Standards<br>Research & Development (multi- & inter-disciplinarity)<br>*Clean energy*<br>*BBP demand (un)certainty*<br>*BBP demand opportunity*<br>*Fossil Resource Scarcity/Finite/Limit*<br>*Biomass Management*<br>*Logistics*<br>Competitiveness<br>***Following four codes…***	*Circular Economy*<br>*Recycling*<br>Sustainable economy/Clean Growth<br>*Environmental Concerns with Resources and BBPS*<br>*Soil*<br>*Water*<br>*Air*<br>*Forests (as environmental concern explicitly)*<br>*Agricultural land (as environmental concern explicitly)*<br>*Arable Land (as environmental concern explicitly)*<br>*Grassland (as environmental concern explicitly)*<br>*Marine (as environmental concern explicitly)*<br>*Urban (as environmental concern explicitly)*<br>*Pollution*<br>*Pesticides*<br>*Plastic/Microplastic pollution*<br>*Land competition*<br>*Environmental Sustainability*<br>*Sustainable resource management*<br>*Sustainable/Low carbon future*<br>*Common Pool Resources*<br>*Standards*<br>*Biosafety*<br>*Ecosystem health and services*<br>*Ecosystem functions & services*<br>*Ecosystem health*<br>*Climate Change adaptation*<br>*Climate threats*<br>*Ocean acidification*<br>*Sea level rise*<br>*Frost*<br>*Heat*<br>*Salinity*

Research-Industry Cooperation<br><br>***Following four codes…***<br>*New products/technologies/ production techniques*<br>*New Technologies*<br>*New Products*<br>*New Processes*<br>***ONLY included if coded in combination with subsequent sectors***<br>*Manufacture of basic pharmaceutical products*<br>*Manufacture of chemicals and chemical products*<br>*Cosmetics*<br>*Medicines/Pharmaceuticals*<br>*Biocides (biological pesticides)*<br>*Biofuels*<br>*Human health and social work activities*<br>*Natural sciences Research and experimental development*	*New products/technologies/ production techniques*<br>*New Technologies*<br>*New Products*<br>*New Processes*<br>***ONLY included if coded in combination with subsequent sectors***<br>*Agriculture, forestry and fishing*<br>*Manufacturing*<br>*Bioenergy*<br>*Industrial inputs*<br>*Food Products*<br>*Wood Products*<br>*Biomass types [all biomass types]*<br>*Organic fertilizers*<br>*Water supply; sewerage, waste management and remediation act*	*Dry Climate*<br><br>*Desertification*<br><br>*Wildfire*<br><br>*Droughts*<br><br>*Rainfalls/Flooding*<br><br>Clean energy<br>*Indigenous Knowledge*<br>*Indigenous Rights*<br>*Small forest owners*<br>*Small-holder-farmer participation/inclusion/farm diversity*<br>*Culture/Cultural Capacity*<br>*Organic fertilizers*<br>*Biodiversity*<br>*Habitat*<br>*Species*<br>*Genetic*<br>Coastal Development<br>Regional Development/Growth<br>Rural Development<br>*Contained Systems*<br>*Water supply; sewerage, waste management and remediation act*<br>*Ethical concerns*<br>Supply (In-)Dependence on Other Countries<br>*Decentralized decision making*<br>*Bottom-Up Decision Making*<br>*Grassroot-stakeholder consultation*

Appendix S6: Text share of goal categories across income groups (in percent)

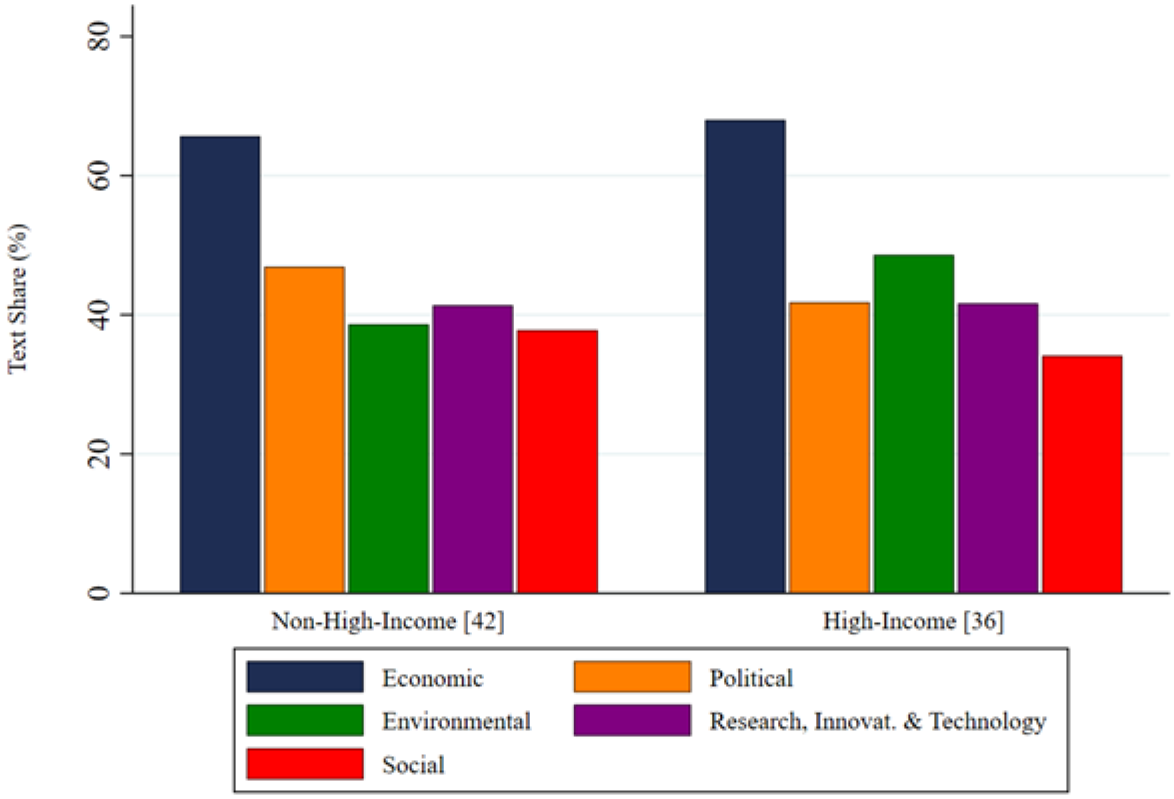

Note: Numbers in square brackets represent the number of included countries per income group.

Appendix S7a: 20 most frequent Economic goal codes by sub-category

Sub-Category	Code Name	Text Share in Economic Category (in percent)
Basic Goods & Services Security	Energysecurity	8.30
	Foodsecurity	5.32
Biomass Management	BiomassSupplyEconomicO	8.32
	BiomassSupplyuncertain	4.95
Business Environment	Incentives	8.13
	NewBusinesses	6.08
	Businessclimategeneric	4.74
Cooperation	PublicPrivateCooperati	5.15
Economic Development/Growth (broadly speaking)	EconomicDevelopmentGro	12.88
Employment	Employment	6.64
Industrial Sector Development	IndustrialSectorDevelopment	12.91
Logistics/Infrastructure	LogisticsInfrastructur	8.33
Market Development	Commercialisation	10.22
	ConsumerBehavior	4.65
	DomesticMarketCreation	4.48
Primary Sector Development	AgriculturalDevelopmen	4.32
Regional Development	RuralDevelopment	4.73
Sustainable Economy	SustainableeconomyClea	8.49
	BioeconomicTransformat	6.66
	CircularEconomy	5.59

Appendix S7b: Frequency of Political goal codes by sub-categories

Sub-Category	CodeName	Text Share in Political Category (in percent)
Governance	StatecapacityforMonitoring	10.67
	InterAuthorityCoordina	10.16
	TopicBroadCrossstakeho	8.57
	Institutions	6.18
	StrategicCapacity	5.38
	GoodGovernance	3.57
	Bureaucracyandadminist	2.28
	TopicGrassrootstakehol	1.22
	DiversityAcknowledgeme	1.09
	TopicBottomUpDecisionM	0.60
	TopicDecentralizeddeci	0.35
	PoliticalInstabilityWa	0.12
	Corruption	0.04
International Cooperation	InternationalCooperati	9.83
	InternationalHarmoniza	6.79
International Political Competition	SupplyInDependenceonOt	5.32
	NationalSupraRegionalR	5.11
	LevelPlayingFieldinTra	2.30
	GeopoliticalOutcomeEqu	0.34
	InternationalPolCompe	0.08
	GeopoliticalProcessEqu	0.00
Regulation	NationalRegulatorysubs	13.82
	NationalRegulatoryunce	3.31
	StateInterventioninEco	1.24
	UrgencyandMomentumforp	1.13
	Recognitionofperversin	1.04

Appendix S7c: 20 most frequent Environmental goal codes by sub-category

**Sub-Category**	**Code Name**	**Text Share in Environmental Category (in percent)**
Sustainable Economy	Sustainable economy	12.84
	Bioeconomic Transformation	10.50
	Circular Economy	7.94
	Recycling	5.08
	Green Economy	2.96
	Blue Economy	2.69
Environmental Sustainability	Sustainable resource management	25.90
	Sustainable Low carbon future	4.32
Environ. Concerns reg. Resources& Bio-based Production Systems	Forests as environmental aim	4.08
	Pollution	3.76
	Water	3.27
	Soil	2.90
	Land competition	2.88
Ecosystem Health & Services	Ecosystem functions and services	3.85
Climate Change	Climate Change mitigation	11.23
	Climate Change adaptation	2.43
Clean Energy	Clean energy	7.37
Biosafety	Biosafety	5.93
Biodiversity	Biodiversity	11.61
-	Environmental	19.23

Appendix S7d: Frequency of Research, Innovation and Technology codes by sub-categories

**Sub-Category**	**Code Name**	**Text Share in Research, Innovation & Technology Category (in percent)**
Biotechnology (explicit)	Biotechnologyexplicit	23.42
	Bioprospecting	2.42
Digitalisation	Digitalisation	4.70
Innovation capacity	Innovationcapacity	19.71
Intellectual Property (Rights)	IntPropRightTopic	2.26
Knowledge-based Bioeconomy (explicit)	KnowledgebasedBioecono	2.13
	IndigenousKnowledge	1.28
New products/technologies/production techniques	NewTechnologies	23.13
	NewProducts	8.41
	Newproductstechnologie	5.83
	NewProcesses	5.54
Research & Development (multi- & inter-disciplinarity	ResearchDevelopmentmul	40.02
Skilled Labor	EducationalTrainingSch	10.01
	SkilledLabour	9.46
Technological (Un)Certainty/Gap	TechnologicalUnCertain	2.92

Appendix S7e: 20 most frequent Social goal codes by sub-category

Sub-Category	Code Name	Text Share in Social Category (in percent)
-	Social	13.21
Accessibility	EnergyInaccessibility	2.50
	MedicinesHealthSystemI	2.22
Behavioral Dynamics	ConsumerBehavior	8.05
	Producerbehavior	3.95
	SocialAcceptanceofbiob	2.95
	Ethicalconcerns	2.26
	SocialAcceptanceofBBPS	1.94
Culture	IndigenousKnowledge	1.94
	CultureCulturalCapacit	1.70
Employment	Employment	14.01
	HighValueJobs	0.87
Equality	Gender	2.26
	Equality	1.81
	UrbanRural	1.49
Human Health	HumanHealth	8.02
	Communicablediseases	2.88
Public understanding/knowledge of bioeconomy	Publicunderstandingkno	10.17
Quality of life/Societal well-being	QualityoflifeSocietalw	10.86
	PovertyReduction	2.56

Appendix S8a: The relative salience of codes making up the Bio-Resource Vision category

**Codes**	**Text share (in percent) of text coded as bio-resource vision**
ResearchDevelopmentmul	26.8
ag3_ec_BiomassManageme	17.7
EconomicDevelopmentGro	13.6
IndustrialManufacturin	12.7
Commercialisation	9.9
NeWProdEtcBiores	9.8
SustainableeconomyClea	9.4
Energysecurity	8.1
LogisticsInfrastructur	7.8
ag4_Competitiveness	6.9
Employment	6.8
ag3_ec_Productivity	5.9
CircularEconomy	5.6
GoalBiores	5.0
RuralDevelopment	4.7
ag3_ec_PrimarySectorDe	4.5
AgriculturalDevelopmen	4.4
Cleanenergy	3.8
Recycling	3.4
InternationalCompetiti	3.1
ag4_Biorefineries	3.0
RegionalDevelopmentGro	2.3
CoopOfCompaniesin	1.9
CoopOfCompaniesac	1.8
Standards	0.9
BBPdemandopportunity	0.8
WaterSecurity	0.7
BBPdemanduncertainty	0.7
Agroprocessing	0.6
ForestEconomy	0.2
FossilResourceScarcity	0.2
CommonPoolResources	0.2
CoastalDevelopment	0.2

Appendix S8b: The relative salience of codes making up the Bio-Technology Vision category

Codes	Text share (in percent) of text coded as bio-technology vision
ResearchDevelopmentmul	36.0
Biotechnologyexplicit	21.3
EconomicDevelopmentGro	20.8
Innovationcapacity	15.4
Commercialisation	14.6
Employment	12.1
NeWProdEtcBiotech	7.5
InternationalCompetiti	4.7
ag4_AgriculturalProduc	4.1
Digitalisation	4.0
ResearchIndustryCooper	2.9
Clusters	2.8
GoalBiotech	2.4
Bioprospecting	2.2
KnowledgebasedBioecono	1.9
MedicinesHealthSystemI	1.8
IntPropRightTopic	1.8
Biotechnologyfirms	1.2
HighValueJobs	1.1
Anchorcompanies	0.4
Cancer	0.2
HIVAIDs	0.1
Tuberculosis	0.1
Malaria	0.1
Diabetes	0.1
Poliomyelitis	0.1
CyberSecurity	0.0
Arthritis	0.0
Biomimicry	0.0
Cardiovasculardisease	0.0
Obesity	0.0
MetabolicSyndrome	0.0

Appendix S8c: The relative salience of codes making up the Bio-Ecology Vision category

Codes	Text share (in percent) of text coded as bio-ecology vision
ag3_en_EnvironmentalSustain	32.5
ag3_en_EnvConcernsReso	16.7
SustainableeconomyClea	13.9
ag3_en_Biodiversity	12.7
RuralDevelopment	8.7
GoalBioeco	8.4
CircularEconomy	7.7
Cleanenergy	5.9
SupplyInDependenceonOt	5.6
Recycling	5.4
ag3_en_EcosystemHealth	5.1
Biosafety	4.4
ag3_so_Inequality	4.1
ag4_Pollution	4.0
RegionalDevelopmentGro	3.6
ag3_so_Culture	3.2
Smallholderfarmerparti	2.5
ClimateChangeadaptatio	2.3
Landcompetition	2.1
Ethicalconcerns	2.0
TopicGrassrootstakehol	1.9
IndigenousKnowledge	1.8
NeWProdEtcBioeco	0.6
ag4_Climatethreats	0.6
TopicDecentralizeddeci	0.3
TopicBottomUpDecisionM	0.3
ag3_so_HumanRights	0.2
CoastalDevelopment	0.2
Smallforestowners	0.0

Appendix S9: Share of vision types in goal-related text share of bioeconomy strategies (in percent)

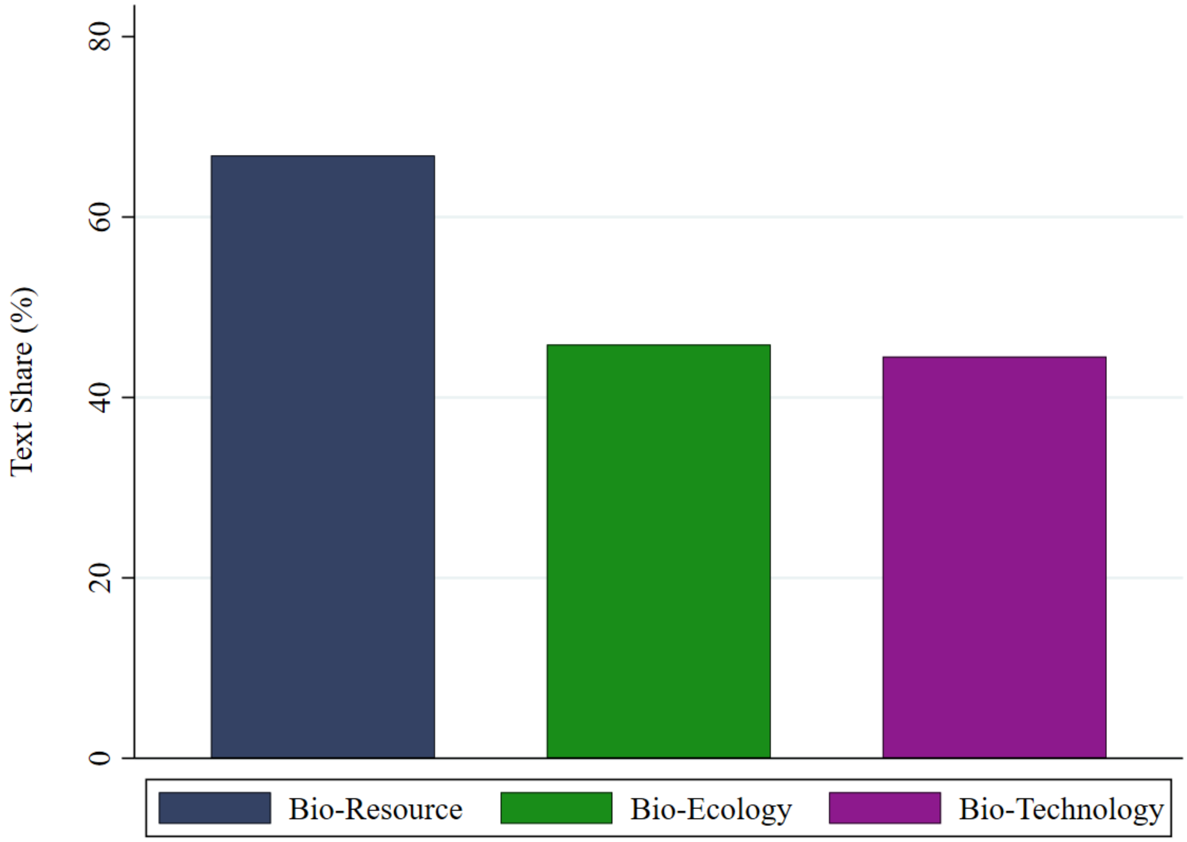

Appendix S10: Share of vision types in goal-related text share compared between the wide and narrow coding frameworks (in percent)

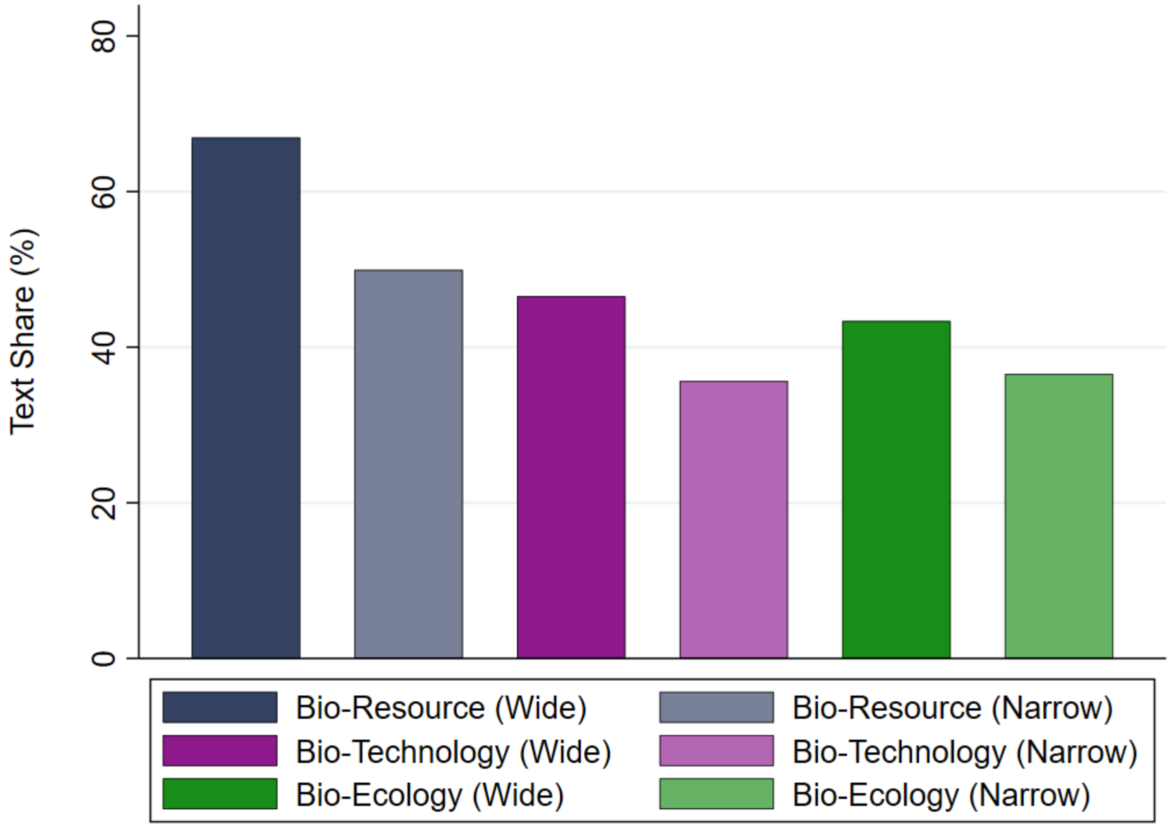

Appendix S11: Scattering of countries and document types within bioeconomy visions for the narrow coding framework

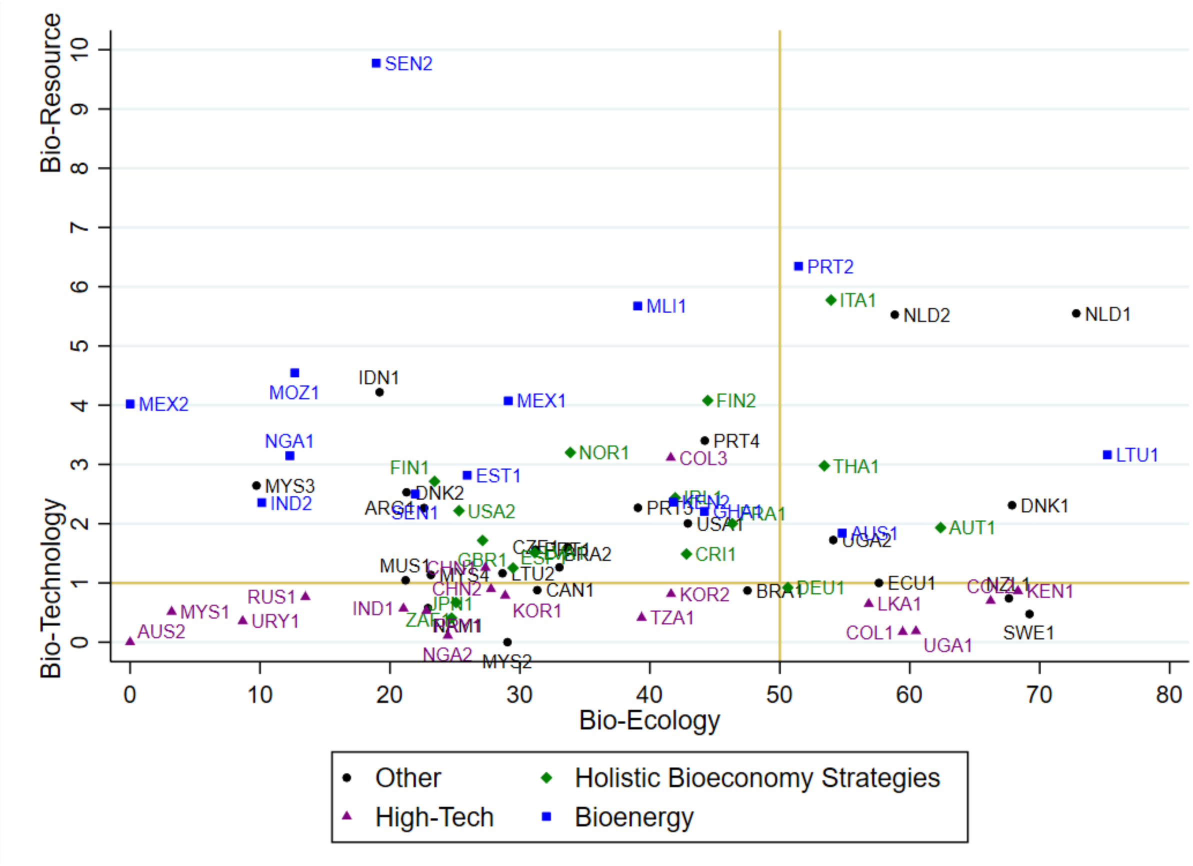

Appendix S12: Salience of visions and goal categories by document types and country (text shares in % of goal-coded text)

Country	Country Code	Document Name	Year	Document Character	Bio-Resource		Bio-Technology		Bio-Ecology		Econ-omic	Environ-mental	Re-search	Pol-itical	Social
					W.	***N.***	***W.***	***N.***	***W.***	***N.***					
**Argentina**	ARG1	Bioeconomía Argentina	2016	Bioeconomy	91	79	63	35	36	23	86	36	62	70	49
**Australia**	AUS1	Opportunities for Primary Industris in The Bioenergy Sector: National Research, Development and Extension Strategy Workplan	2014	Bioenergy	90	86	47	47	74	55	89	59	44	71	34
**Australia**	AUS2	Biotechnology and agriculture in Australia: policy snapshot	2018	High-Tech	45	0	45	45	0	0	55	0	45	55	45
**Austria**	AUT1	Bioeconomy a strategy for Austria	2019	Bioeconomy (holistic)	80	60	44	31	68	62	70	68	41	24	40
**Brazil**	BRA1	Estrategia Nacional de Ciencia, Technologia e Inovacao	2016	Research & Innovation	85	56	77	64	84	48	74	73	83	55	32
**Brazil**	BRA2	Plano de Ação em Ciência, Tecnologia e Inovação em Bioeconomia	2018	Research & Innovation	76	60	50	47	38	33	68	43	56	37	25
**Brazil**	BRA3	Plano Decenal de Expansão de Energia 2029	2020	Bioenergy	22	22	4	0	0	0	22	60	0	70	24
**Canada**	CAN1	Canada's Bioeconomy Strategy	2019	Bioeconomy	65	42	57	48	32	31	78	42	45	56	12
**China**	CHN1	13th FYP on Bioindustry Development	2016	High-Tech	84	77	66	61	27	27	79	32	64	34	36
**China**	CHN2	13th FYP for Strategic Emerging Industries	2016	High-Tech	69	69	81	76	28	28	69	42	76	10	0
**Colombia**	COL1	Política par el Desarrollo Comercial de la Biotecnología a partir del uso Sostenible de la Biodiversidad	2011	High-Tech	49	10	65	57	59	59	65	59	54	92	11
**Colombia**	COL2	Colombia Bio	2016	High-Tech	53	30	49	43	66	66	28	66	53	39	12
**Colombia**	COL3	Colombia Green Growth Roadmap	2018	High-Tech	49	32	31	10	47	42	72	80	17	57	25
**Costa Rica**	CRI1	National Bioeconomy Strategy – Costa Rica 2020 – 2030	2020	Bioeconomy (holistic)	62	52	41	35	55	43	78	66	51	20	33

**Czech Republic**	CZE1	Bioeconomy concept in the Czech Republic from the perspective of the Ministry of Agriculture 2019 – 2024	2019	Bioeconomy	57	38	29	24	39	34	56	46	30	30	29
**Denmark**	DNK1	Plan for Growth for Water, Bio & Environmental Solutions	2013	Green Economy	82	65	51	28	68	68	73	68	54	9	23
**Denmark**	DNK2	Denmark as growth hub for a sustainable bioeconomy	2014	Bioeconomy	47	24	25	9	21	21	66	27	17	70	20
**Denmark**	DNK3	Strategy for Circular Economy	2018	Circular Bioeconomy	91	86	16	0	80	79	82	76	10	64	17
**Ecuador**	ECU1	Lineamientos Para El Fomento Del Bioemprendimiento	2019	Biobased Economy	24	16	24	16	58	58	63	62	16	63	28
**Estonia**	EST1	Development Plan on the Promotion of Biomass and Bioenergy Use for 2007–2013	2007	Bioenergy	71	64	32	23	33	26	78	48	31	60	40
**Finland**	FIN1	The Finnish Bioeconomy Strategy	2014	Bioeconomy (holistic)	57	35	38	13	38	23	78	62	19	48	32
**Finland**	FIN2	Competitive Advantage from Clean Food and Responsible Bioeconomy and Circular Economy	2018	Bioeconomy (holistic)	69	52	61	13	51	44	79	62	32	40	33
**France**	FRA1	A Bioeconomy strategy for France (Goals & Action Plan)	2018	Bioeconomy (holistic)	62	51	30	26	56	46	62	55	31	35	35
**Germany**	DEU1	Nationale Bioökonomiestrategie	2020	Bioeconomy (holistic)	68	39	58	43	55	51	49	54	50	42	24
**Ghana**	GHA1	Ghana renewable energy master plan	2019	Bioenergy	65	52	26	24	47	44	57	43	32	43	45
**India**	IND1	National Biotechnology Development Strategy 2015 - 2020	2015	High-Tech	91	49	90	86	25	21	49	14	83	36	69
**India**	IND2	National Policy on Biofuel	2018	Bioenergy	77	70	37	30	16	10	72	18	33	16	30
**Indonesia**	IDN2	Bioenergy Policies and Regulation in Indonesia	2014	Bioenergy	75	75	13	0	71	58	60	58	0	38	15
**Indonesia**	IDN1	Master Strategy for Agricultural Development 2015 - 2045	2014	Biobased Economy	60	47	24	11	29	19	71	22	16	55	42
**Ireland**	IRL1	National Policy Statement on the Bioeconomy	2018	Bioeconomy (holistic)	54	37	32	15	49	42	53	45	15	46	25

**Italy**	ITA1	BIT II - Bioeconomy in Italy: A new bioeconomy strategy for a sustainable Italy (including Action Plan)	2019	Bioeconomy (holistic)	68	60	22	10	60	54	67	55	25	39	32
**Japan**	JPN1	Bio Strategy Japan 2019/2020	2019	Bioeconomy (holistic)	65	26	71	64	28	25	58	25	66	19	39
**Kenya**	KEN1	A National Biotechnology Development Policy	2006	High-Tech	89	79	92	91	72	68	84	58	92	33	66
**Kenya**	KEN2	Strategy for Developing the Bio-diesel Industry in Kenya	2008	Bioenergy	80	68	34	29	53	42	73	37	23	43	49
**Latvia**	LVA1	Latvian Bioeconomy Strategy 2030 (LI-BRA)	2017	Bioeconomy (holistic)	55	43	45	28	40	31	71	39	33	40	36
**Lithuania**	LTU1	National Renewable Energy Action Plan	2010	Bioenergy	79	79	25	25	79	75	45	73	37	38	38
**Lithuania**	LTU2	Action Plans on Biomass Energy, Biomolecular Biotechnologies and Biorefineries promotion as part of the Lithuanian Smart Specialisation Programme	2014	Biobased Economy	77	54	58	46	29	29	58	22	81	24	28
**Malawi**	MWI1	Malawi Biomass Energy Strategy	2009	Bioenergy	42	35	14	0	21	19	47	21	4	76	25
**Malaysia**	MYS1	“National Biotechnology Policy” (2005-2020)	2005	High-Tech	62	32	69	61	3	3	59	3	60	39	30
**Malaysia**	MYS2	Malaysia´s National Blue Ocean Shift	2009	Blue Economy	43	0	66	23	29	29	68	14	23	23	38
**Malaysia**	MYS3	National Biomass Strategy 2020: New wealth creation for Malaysia’s biomass industry (Version 2.0)	2013	Biobased Economy	87	87	46	33	10	10	94	6	32	37	29
**Malaysia**	MYS4	Bioeconomy Corportion Bioeconomy Transformation Programme (BTP)<br><br>I believe there are more recent reports as well.	2015	Bioeconomy	71	45	66	40	39	23	86	49	40	41	51
**Mali**	MLI1	Stratégie Nationale pour le Développement des Biocarburants en Mali & Loi pour l'etabilissement de l'Agence Nationale de Développement des Biocarburants	2008	Bioenergy	78	74	27	13	49	39	82	44	26	55	60
**Mauritius**	MUS1	The Ocean Economy	2013	Blue Economy	47	19	34	19	29	21	66	51	23	63	24
**Mexico**	MEX1	Estrategia Intersecretarial de los Bioenergéticos	2009	Bioenergy	89	70	19	17	52	29	82	61	10	44	54

**Mexico**	MEX2	Transition Strategy to Promote the Use of Cleaner Technologies and Fuels	2020	Bioenergy	44	44	11	11	33	0	44	42	30	91	64
**Mozambique**	MOZ1	Política e Estratégia de Biocombustiveis	2009	Bioenergy	55	46	22	10	28	13	61	15	13	49	38
**Mozambique**	MOZ2	Mozambique Biomass Energy Strategy	2012	Bioenergy	58	45	28	3	38	35	70	39	6	51	43
**Namibia**	NAM1	The National Programme on Research, Science, Technology & Innovation	2014	Research & Innovation	76	39	77	68	33	23	63	28	73	44	36
**Netherlands**	NLD1	Strategic Biomass Vision for the Netherlands towards 2030	2016	Biobased Economy	81	77	27	14	73	73	78	76	20	44	4
**Netherlands**	NLD2	The position of the bioeconomy in the Netherlands	2018	Bioeconomy	85	72	25	13	64	59	77	64	39	20	33
**New Zealand**	NZL1	Primary Sector Science Roadmap - Te Ao Turoa	2017	Research & Innovation	73	37	67	50	72	68	64	62	73	21	50
**Nigeria**	NGA1	Nigeria Biofuels policy and incentives	2007	Bioenergy	95	79	43	25	16	12	100	16	30	29	18
**Nigeria**	NGA2	National Biotechnology Development Agency Bill	2015	High-Tech	52	7	69	65	24	24	30	26	73	68	14
**Norway**	NOR1	Familiar resources – undreamt of possibilities. Government Bioeconomy Strategy	2016	Bioeconomy (holistic)	69	58	23	18	38	34	81	52	24	31	17
**Paraguay**	PRY1	Politica y Programa Nacional de Biotecnoloía Agropecuaria y Forestal del Parauay (2011)	2011	High-Tech	64	44	86	83	32	23	69	16	87	62	62
**Portugal**	PRT1	National Ocean Strategy 2013-2020	2013	Blue Economy	39	19	29	12	42	31	51	50	21	79	41
**Portugal**	PRT3	Circular Economy Action Plan	2017	Circular Bioeconomy	49	42	27	18	40	39	62	47	23	66	31
**Portugal**	PRT2	National Plan for the Promotion of Biorefineries	2017	Bioenergy	84	79	17	12	56	51	83	67	8	28	27
**Portugal**	PRT4	Portuguese Bioeconomy Strategy Roadmap	2019	Blue Economy	69	60	18	18	48	44	69	35	18	44	25
**Russia**	RUS1	Comprehensive Program and Roadmap for the Development of Biotechnology in Russia by 2020	2012	High-Tech	67	56	76	74	20	13	74	23	68	22	17
**Senegal**	SEN1	Biofuels Jatropha Program 2007-2012	2007	Bioenergy	100	100	70	40	52	22	100	0	0	55	92

**Senegal**	SEN2	Lettre de Politique de Développement du Secteur de L'Energie	2008	Bioenergy	69	63	17	6	49	19	73	19	0	31	55
**South Africa**	ZAF1	The Bio-economy Strategy	2013	Bioeconomy (holistic)	73	46	78	69	31	25	72	28	67	41	41
**South Korea**	KOR1	Biovision 2016 - For Building a Healthy Life and a Prosperous Bioeconomy	2006	High-Tech	75	58	74	74	37	29	70	18	74	21	48
**South Korea**	KOR2	Biotechnology in Korea	2018	High-Tech	82	65	82	80	42	42	41	11	80	14	62
**Spain**	ESP1	The Spanish Bioeconmy Strategy 2030 Horizon	2016	Bioeconomy (holistic)	68	42	47	34	40	29	81	54	60	33	64
**Sri Lanka**	LKA1	National Biotechnology Policy	2010	High-Tech	59	50	79	77	61	57	60	47	76	27	39
**Sweden**	SWE1	Swedish Research and Innovation Strategy for a Bio-based Economy	2012	Research & Innovation	80	35	80	74	69	69	52	81	80	44	69
**Tanzania**	TZA1	National Biotechnology Policy	2010	High-Tech	63	36	89	88	45	39	52	50	89	77	62
**Tanzania**	TZA2	Biomass Energy Strategy (BEST) Tanzania	2014	Bioenergy	35	23	17	1	48	47	39	47	6	85	10
**Thailand**	THA1	Bio-Circular-Green Economy (BCG) in Action: The new Sustainable Growth Engine	2019	Bioeconomy (holistic)	78	73	39	25	59	53	73	50	36	39	44
**Uganda**	UGA1	National Biotechnology and Biosafety Policy	2008	High-Tech	42	12	64	62	63	60	51	45	69	30	46
**Uganda**	UGA2	Biomass Energy Strategy (BEST)	2018	Biobased Economy	60	51	33	30	54	54	59	50	46	53	54
**United Kingdom**	GBR1	Growing the Bioeconomy	2018	Bioeconomy (holistic)	69	41	47	24	37	27	74	41	36	19	23
**United States**	USA2	Strategic plan for thriving and sustainable bioeconomy	2016	Bioeconomy (holistic)	71	61	37	27	28	25	73	52	46	33	29
**United States**	USA1	Federal Activities report on the bioeconomy	2016	Bioeconomy	79	55	50	27	52	43	78	60	46	73	50
**Uruguay**	URY1	Plan Sectorial de Biotechnología 2011-2020	2012	High-Tech	53	32	89	89	15	9	60	17	82	53	21

Note: "W" and "N" stand for the wide and narrow scores of the bioeconomy vision coding frameworks.
